# Fear of cancer recurrence and PSA anxiety in patients with prostate cancer: a systematic review

**DOI:** 10.1007/s00520-022-06876-z

**Published:** 2022-02-01

**Authors:** Callum James, Oliver Brunckhorst, Omar Eymech, Robert Stewart, Prokar Dasgupta, Kamran Ahmed

**Affiliations:** 1 MRC Centre for Transplantation, King’s College London, King’s Health Partners, Guy’s Hospital Campus, 5th Floor Southwark Wing, London, SE1 9RT UK; 2 King’s College London Institute of Psychiatry, Psychology and Neuroscience, London, UK; 3 South London and Maudsley NHS Foundation Trust, London, UK; 4 Urology Centre, Guy’s and St. Thomas’ NHS Foundation Trust, King’s Health Partners, London, UK; 5 Department of Urology, King’s College Hospital, London, UK

**Keywords:** Cancer, Fear of recurrence, Mental health, Mental wellbeing, Oncology, Prostate cancer, PSA anxiety

## Abstract

**Purpose:**

The impact of prostate cancer on the mental wellbeing of patients is increasingly being appreciated. Two important aspects of this include fear of cancer recurrence (FCR) and prostate-specific antigen (PSA) anxiety. However, their prevalence, severity and associating factors remain poorly understood. Therefore, this review aims to evaluate the current evidence for the prevalence, severity and associating features of PSA anxiety and FCR.

**Methods:**

A systematic search of MEDLINE, EMBASE and PsycINFO databases was conducted by two independent reviewers. Observational studies measuring FCR and PSA anxiety in prostate cancer using validated measures were included. Outcome measures were prevalence of significant levels, mean scores and significant correlations of FCR and PSA anxiety scores with patient, disease, treatment or other mental health and quality of life outcomes.

**Results:**

One thousand one hundred forty-eight individual records underwent screening with 32 studies included. Median prevalence of significant FCR and PSA anxiety was 16% and 22% respectively across all studies. Longitudinal studies demonstrated severity of both symptoms peaks at diagnosis, with little variability, even several years following this. Evaluating associating factors revealed younger age, generalised quality of life and mental health symptoms to be important factors for both outcomes. Few studies evaluated associations and differences between other patient, disease and treatment characteristics.

**Conclusion:**

FCR and PSA anxiety are prominent symptoms for prostate cancer patients and importantly when present, are associated with poorer quality of life and mental health symptoms. Screening for these constructs and referral to appropriate services should form part of routine follow-up care.

**Supplementary Information:**

The online version contains supplementary material available at 10.1007/s00520-022-06876-z.

## Background

Prostate cancer remains the most commonly diagnosed male cancer in Europe with increasing 10-year survival rates now reaching 80% [1]. Growing global incidences have been attributed to wider utilisation of prostate-specific antigen (PSA) screening resulting in more localised prostate cancer diagnoses. Combining these factors has led to a belief that prostate cancer can often be classified as a chronic condition [2], and as such, issues beyond the pure physical health of the patient are prominent. The psychological impact of prostate cancer is now increasingly recognised [3]. However, it is important to look beyond formal mental health disorders when considering the mental wellbeing of individuals, with other distinct constructs being important for mental wellbeing [4]. Fear of cancer recurrence (FCR) is one of these, being reported as the most common unmet cancer need at 5 years post survival [5]. A common definition describes this as the “Fear, worry, or concern relating to the possibility that cancer will come back or progress” [6]. Despite frequently being neglected during care, FCR is a known significant factor in both physical and mental health for prostate cancer survivors [7]. FCR has already been widely explored in other cancers including breast and testicular cancer where it is not only demonstrated to be present in different levels in 30% of survivors, but has additionally been shown to have a significant correlation with poorer general quality of life and other wellbeing issues such as self-esteem [8].

PSA testing and monitoring poses another distinct source of anxiety for patients [7], with PSA anxiety (previously described as PSAdynia) being a unique problem in prostate cancer. This is commonly seen as the “state of physical or emotional distress due to an elevated PSA level” [9]. It can have a major impact on patients’ overall wellbeing, affecting how they view their symptoms. Both FCR and PSA anxiety are reported as two key factors for distress in patients and due to the usage of PSA testing as an investigation for recurrence have been demonstrated to be closely interlinked [10]. Higher PSA levels lead to higher levels of PSA anxiety and higher levels of generalised cancer-related anxiety [11]. However, despite the importance of FCR and PSA anxiety to mental wellbeing for prostate cancer patients, there remain varied findings in the literature as to their prevalence, severity and progression during the disease process. Furthermore, little is known about which associative factors and modulators are related to these symptoms and the impact they can have on a patient’s health. There are very heterogenous ideas over which factors act as modulators of FCR and PSA anxiety or not. Therefore, this systematic review aims to:

1. Identify the prevalence, severity and progression of FCR and PSA anxiety symptoms in prostate cancer patients.
2. Evaluate the evidence for the association between patient, disease or treatment characteristics and FCR and PSA anxiety symptoms.
3. Assess the relationship between FCR and PSA anxiety and other psychological and quality of life outcomes and their impact on mental wellbeing.

## Methods

This review was conducted adhering to the synthesis without meta-analysis (SWiM), the Preferred Reporting Items for Systematic reviews and Meta-Analyses (PRISMA) and PRISMA-literature search extension (PRISMA-S) guidelines [12–14]. A priori protocol was registered on the Prospero database (CRD42020225154).

### Study eligibility criteria

Inclusion criteria were observational studies with data available on FCR or PSA anxiety severity or prevalence in a prostate cancer sample. We included both cross-sectional and longitudinal cohort study (retrospective and prospective) designs. Participants undergoing any management option were included. Studies required the use of a previously validated psychometric tool to measure the outcomes of FCR and PSA anxiety, with prior evidence in a cancer population.

We excluded all interventional studies, reviews and opinion articles. Conference abstracts with insufficient information for evaluation of study quality and papers without an English translation were also excluded. Where a study sample included a mixed cancer population, this was excluded if individual results for the prostate cancer population were not available. Duplicate datasets were excluded with the most up to date or comprehensive data selected. Lastly, we excluded studies if non-validated outcome measures were used to record FCR or PSA anxiety or if they measured other constructs of mental wellbeing such as generalised distress or anxiety.

### Information sources and search

A systematic literature search was conducted on the MEDLINE (via Pubmed), EMBASE and PsycINFO (both via OvidSP) databases from inception to 24/08/2021. The search strategy was piloted prior to use and included a mixture of key words, MeSH terms and commonly used abbreviations relating to prostate cancer, FCR and PSA anxiety (online resource 1). Grey literature was searched through conference abstracts on EMBASE and potentially relevant ongoing studies on clinicaltrials.gov; however, no relevant ongoing studies were identified. Lastly, we conducted a reference review of included articles.

### Study selection

Studies were independently screened by two reviewers (CJ and OB) through title, abstract and subsequently full-text review against inclusion criteria. Rayyan software was used to manage and screen identified studies [15]. Discrepancies during screening were discussed, until there was 100% agreement. Lastly, studies deemed as high risk of bias according to our study quality assessment were excluded from final inclusion at this stage. All studies excluded at the full-text stage are listed in online resource 2.

### Data collection and data items

Two reviewers (CJ and OB) independently extracted data onto a pre-defined and piloted extraction sheet. Study characteristics extracted from included studies were author, study design, country of study, year of publication, psychometric tool utilised to assess outcome, cut-off used for caseness of outcome and time since diagnosis at data collection. Additionally, we extracted participant characteristics such as age, demographics and treatment received for their prostate cancer. Our outcome measures of interest extracted included data relating to the prevalence of significant levels of FCR or PSA anxiety, raw number of patients meeting cut-off, mean scores of utilised measures for FCR and PSA anxiety, correlations between patient factors and FCR/PSA anxiety as measured by correlation scores. In addition, both the severity and progression over time of each construct were assessed using the most commonly used scale to measure each construct. The full study characteristics are provided in online resource 3.

### Summary measures and synthesis of results

A meta-analysis was found to be unfeasible due to heterogenous designs of studies and reporting of outcomes. Therefore, a structure qualitative synthesis was conducted. Studies were first grouped through the constructs they were measuring (FCR or PSA anxiety) and subsequently through the aim of the review they addressed. Descriptive statistics were utilised to describe some outcomes of interest including prevalence of significant symptoms, mean and median scores. Due to heterogeneity of data, vote counting was used to measure the direction of effect, with study risk of bias rating and size of effect used to measure the clinical significance of findings.

### Study quality

Individual study risk of bias evaluation was conducted using the Joanna Briggs Institute (JBI) checklist (online resource 4) for cross-sectional and cohort studies, depending on study design, by two independent reviewers (CJ and OE) [16]. These were selected as they allowed for evaluation of the internal and external validity of observational studies with varying designs. Total scores were calculated with pre-determined cut-offs based around percentage scores. A total score of 0–4 represented a high risk of bias, 5–6 a moderate and > 7 a low risk of bias. Studies with a high risk of bias were subsequently excluded from the review.

## Results

### Study selection and characteristics

**Fig. 1:**
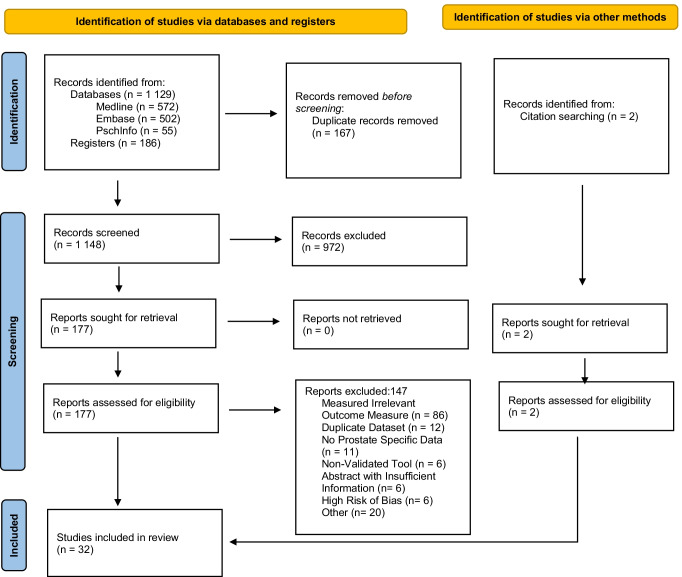
PRISMA flow diagram of study selection. From: Page MJ, McKenzie JE, Bossuyt PM, Boutron I, Hoffmann TC, Mulrow CD, et al. The PRISMA 2020 statement: an updated guideline for reporting systematic reviews. BMJ 2021;372:n71. https://doi.org/10.1136/bmj.n71. For more information, visit http://www.prisma-statement.org/

Post deduplication, 1148 results underwent abstract and title screening, with 177 undergoing full-text review (Fig. 1). After full-text review, six studies were excluded due to high risk of bias, leaving a final 32 studies included. Twenty-seven studies measured FCR (Table 1) with a total of 8715 patients [17–41] and 18 PSA anxiety (Table 2) combining 9953 patients [11, 17–22, 26, 27, 30, 36, 38, 39, 41–45]. Twelve studies measured both FCR and PSA anxiety domains [11, 17–22, 26, 30, 36, 38, 39]. Studies drew populations from varied countries with 13 from Europe, 11 from North America, 6 from Australia and 2 from Asia. Dates of publication ranged from 2003 to 2021, with 18 studies utilising a longitudinal design and 14 a cross-sectional design.

**Table 1:** Fear of cancer recurrence study results

Study	Sample size (*n*)	Outcome measure tool utilised	JBI risk of bias classification	Treatment received	Timepoint	Mean/median outcome measure score	Prevalence of significant FCR (%)
Chien et al. 2018 [36]	48	MAXPC-FOR	Low	RP, RT	Diagnosis	5.88	N/A
					6 weeks	5.69	
					10 weeks	5.17	
					18 weeks	5.04	
					24 weeks	4.89	
Huang et AL. 2014 [30]	254	MAXPC-FOR	Low	RP, RT, BT, orchidectomy	N/A	Early 5.21	N/A
						Local 5.26	
						Advanced 6.27	
Naha et al. 2020 [26]	302	MAXPC-FOR	Low	AS	0 months	3–3.5	N/A
					36 months	3–3.5	
Tavlarides et al. 2013 [21]	365	MAXPC-FOR	Low	RP	12 months	1b	N/A
Alvisi et al. 2020 [39]	236	MAXPC-FOR	Moderate	AS	0 months	0.8	13
					10 months	0.9	16
Anderson et al. 2014 [38]	86	MAXPC-FOR	Moderate	AS	22 months	3.26	8.1
Ettridge et al. 2020 [33]	276	MAXPC-FOR*	Moderate	RP, RT, ADT, AS/WW	6 months	8.6	N/A
					12 months	8.1	
					24 months	9	
Kendel et al. 2016 [29]	Total = 370	MAXPC-FOR	Moderate	AS, RP	4 years (mean)	Total = 3.05a	N/A
	AS = 140					AS = 3.33	
	DAS = 78					DAS = 3.09	
	RP = 152					RP = 2.76	
Mehnert et al. 2007 [27]	197	MAXPC-FOR	Moderate	RP	10 months (mean)	1.2 a	N/A
Mehta et al. 2003 [40]	RP = 326	FCR scale	Moderate	RP, RT, BT	0–6 months	RP = 77, RT = 73, BT = 69	N/A
	RT = 53				6–12 months	RP = 78, RT = 73, BT = 73	
	BT = 140				18–24 months	RP = 78, RT = 71, BT = 71	
Parker et al. 2016 [24]	180	MAXPC-FOR	Moderate	AS	0 months	4.3	N/A
					6 months	3.7	
					12 months	3.3	
					18 months	3	
					24 months	2.6	
					30 months	2.6	
Roth et al. 2003 [41]	385	MAXPC FOR	Moderate	RP, RT, BT, HT, orchidectomy	N/A	N/A	N/A
Touzani et al. 2019 [20]	185	MAXPC-FOR	Moderate	Not stated	Not stated	5.8, 6b	N/A
Ussher et al. 2016 [19]	GB = 119	MAXPC-FOR	Moderate	AS, RP, RT, ADT, HT	GB = 5.9 years (mean)	GB = 4.29	N/A
	H = 224				H = 7.7 years (mean)	H = 3.32	
van den Bergh et al. 2010 [17]	129	MAXPC-FOR	Moderate	AS	6 months	4.2, 4.0b	N/A
					12 months	3.5, 4.0b	
Villa et al. 2015 [22]	207	MAXPC-FOR	Moderate	AS	0 months	3.35	N/A
					19 months	3.1	N/A
Bellizzi et al. 2007 [37]	730	Kornblith’s 5-item FOR scale	Low	RP, RT	0 months	36.1	N/A
					12 months	23.5	
Hart et al. 2014 [31]	92	Kornblith’s 5-item FOR scale	Moderate	RP, RT, BT, HT	1.91 years (mean)	49.2	N/A
Egger et al. 2017 [35]	AS/WW = 63	Kornblith 5-item FOP scale	Moderate	AS/WW	9.8 years (mean)	AS/WW = 71.3	N/A
	RP = 221			RP		RP = 80.5	
	RT = 25			RT		RT = 74.2	
	BT = 32			BT		BT = 81.1	
Eisenberg et al. 2014 [34]	67	5-item FCR scale	Low	RP, RT, HT, AS	Not stated	10.45	4.16
Nilsson et al. 2021 [25]	440	CARQ	Moderate	AS	Not stated	AS = 10.1	AS = 37.1
				RP		RP = 9	RP = 29.4
Van de Wal et al. 2016 [18]	283	Cancer worry scale	Moderate	RP, RT	7 months (mean)	15.8	36
Koch-Gallenkamp 2016 [28]	2162	FOP-Q	Moderate	Not stated	Not stated	24.5	8
Götze et al. 2019 [32]	255	FOP-Q	Low	Not stated	7.5 years (mean)	20.6	4.9
Meissner et al. 2021 [46]	2417	FOP-Q	Low	RP	7 years	21.2	6.5
					16 years	22.2	8.4
Sevier guy et al. [59]	144	FCR7	Low	AS, RP, RT, ADT	6 years (mean)	15.9	N/A
Savard et al. 2018 [23]	263	FCRI	Moderate	Not stated	0 months	15	45
					18 months	11.8	39

a = mean, b = median,* = reverse scale, higher scores = lower fear

*ADT*, androgen deprivation therapy; *AS*, active surveillance; *BT*, brachytherapy; *CARQ*, concerns about recurrence questionnaire; *FACT-P*, functional assessment of cancer therapy prostate; *FCRI*, fear of cancer recurrence inventory; *FOP-Q*, fear of progression questionnaire; *FOR*, fear of recurrence; *GB*, gay and bisexual patients; *H*, heterosexual patient; *HADS*, hospital anxiety and depression score; *HT*, hormone therapy; MAXPCFOR, memorial anxiety scale for prostate cancer fear of recurrence subscale; *RP*, radical prostatectomy; *RT*, radiation therapy; *WW*, watchful waiting

**Table 2:** PSA anxiety study results

Study	Sample size	Outcome measure tool utilised	JBI risk of bias classification	Treatment received	Timepoint	Mean/median outcome measure score	Prevalence of significant PSA anxiety (%)
Chambers et al. 2017 [44]	1,064	MAXPC-PSA	Low	RP, RT, WW, HT, BT	0 years	2.43a	N/A
					6 years	2.08	
Chien et al. 2018 [36]	48	MAXPC-PSA	Low	RP, RT	Diagnosis	1.76a	N/A
					6 weeks	1.38a	
					10 weeks	1.25a	
					18 weeks	1.45a	
					24 weeks	1.24a	
Meissner et al. 2017 [11]	4,719	MAXPC-PSA	Low	RP	11.5 years (mean)	N/A	3
Huang et al. 2014 [30]	254	MAXPC-PSA	Low	RP, RT, BT, orchidectomy	Not stated	Early 0.87a	N/A
						Local 0.63a	
						Advanced 1.36	
Naha et al. 2020 [26]	302	MAXPC-PSA	Low	AS	0 months	0–0.5a	N/A
					36 months	0–0.5a	
Tavlarides et al. 2012 [21]	365	MAXPC-PSA	Low	RP	12 months (mean)	0b	N/A
Alvisi et al. 2018 [39]	236	MAXPC-PSA	Moderate	AS	0 months	0.9a	20.2
					10 months	1.1a	30
Anderson et al. 2014 [38]	86	MAXPC-PSA	Moderate	AS	22 months (mean)	0.45a	1.2
Mehnert et al. 2007 [27]	197	MAXPC-PSA	Moderate	RP	10 months (mean)	0.35a	N/A
Mahal et al. 2015 [43]	ADT = 68	MAXPC-PSA	Moderate	ADT, RT	N/A	N/A	27.9
	RT = 307						15.3
Roth et al. 2003 [41]	385	MAXPC-PSA	Moderate	RP, RT, BT, HT, orchidectomy	N/A	N/A	N/A
Touzani et al. 2019 [20]	185	MAXPC-PSA	Moderate	Not stated	Not stated	1.2a, 0b	N/A
Ussher et al. 2016 [19]	GB = 119	MAXPC-PSA	Moderate	AS, RP, RT, HT, ADT	GB = 5.9 years (mean)	GB = 1.02a	N/A
					H = 7.7 years (mean)	H = 0.27a	N/A
	H = 224						
Van de Wal et al. 2016 [18]	283	MAXPC-PSA	Moderate	RP, RT	7.1 months (mean)	High FCR = 0.7a	N/A
						Low FCR = 0.2a	N/A
van den Bergh et al. 2015 [17]	129	MAXPC-PSA	Moderate	AS	6 months	0.3a	N/A
					12 months	0.3a	
Villa et al. 2010 [22]	207	MAXPC-PSA	Moderate	AS	0 months	0.68a	N/A
					19 months	0.54a	
Clark et al. 2006 [42]	235	Previously validated measure (0–100) 100	Low	RP, RT, AS/WW, ADT	6 years (mean)	65.91a	N/A
Dowrick et al. 2018 [45]	540	Clark cancer worry PSA cancer subscale	Moderate	RP	1 year (mean)	55.6b	N/A

a = mean; b = median; *ADT*, androgen deprivation therapy; *AS*, active surveillance; *BT*, brachytherapy; *FCR*, fear of cancer recurrence; *GB*, gay or bisexual; *HT*, hormone therapy; *MAXPC-PSA*, memorial anxiety scale for prostate cancer prostate-specific antigen subscale; *RP*, radical prostatectomy; *RT*, radiation therapy; *WW*, watchful waiting

### Fear of cancer recurrence

#### Prevalence

Nine studies measured prevalence of significant FCR ranging from 4.1 to 45% (median: 13%, IQR = 29.3) in a total of 6210 patients [18, 23, 25, 28, 32, 34, 38, 39, 46]. Only two longitudinal studies measured the evolution of significant prevalence over time, with one demonstrating a small decrease from 45% at treatment to 39% by 18 months suffering with high FCR [23]. The other showed prevalences of high FCR at 7 years post radical prostatectomy at 5% increasing to 7% at 16 years post surgery. Nilsson et al. [25] found higher prevalence of FCR in active surveillance (AS) patients compared to radical prostatectomy (RP). The prevalence of low risk of bias studies had a median of 5.7% (IQR = 2.92) [32, 34, 46] compared to a median prevalence of 29.4 (IQR = 27.5) for moderate risk of bias studies [18, 23, 25, 28, 38, 39].

#### Severity and progression over time

There were seven different measures used to measure FCR, with the memorial anxiety prostate cancer fear of recurrence (MAXPC FoR) subscale most commonly used with 12 out of the 27 studies using it. This is scored out of 12 with higher scores signifying higher levels of FCR. Average scores of 1.5 on each question or a combined score of > 6 have been previously classified as significant [20]. Five studies measured FCR levels with this subscale at diagnosis with mean scores ranging from 0.8 to 5.88 (median = 3.35) and no mean scores at diagnosis above the cut-off [22, 24, 26, 36, 39]. Eight studies measured FCR scores at 6 months to a year after diagnosis with mean scores ranging from 1 to 4.89 (median = 3.6, IQR = 3.8). Lastly, seven evaluated FCR levels at greater than a year post diagnosis with mean scores ranging from 2.6 to 4.29 (median = 3.1, IQR = 0.42) [19, 22, 24, 26, 29, 33, 38]. Nine studies evaluating the progression of FCR severity over time demonstrated scores to be highest at diagnosis with a decrease over time [17, 19, 22–24, 33, 36, 37, 39]. This decrease was however often very small with only a only a single study identifying this change to be statistically significant between 0 and 2 months [23].

#### Predictors of fear of cancer recurrence

Few factors associated with FCR were consistently evaluated. Most studies measured FCR for prostate cancer patients as a whole, with few distinguishing between treatment groups. Kendel et al. [29] however compared patients on active surveillance, radical prostatectomy and patients who discontinued active surveillance, identifying active surveillance patients specifically to possess higher levels of FCR. Conversely, Nilsson et al. found RP patients to have higher FCR than AS patients [25] and Mehta et al. found [40] higher levels of FCR in radiotherapy than RP. Separately with a metastatic disease at diagnosis [30] was also found to be associated with higher FCR as compared to those with localised cancer diagnosis. Evaluating patient characteristics, higher levels of FCR were seen in gay or bisexual patients in two studies [19, 31]. Additionally, seven studies found a negative association between age and FCR levels [18, 20, 25, 30, 36, 38, 41], implying younger patients display higher levels of FCR.

#### Relationship to other outcomes

FCR was consistently associated with other quality of life and mental health measures (Table 3). In particular, FCR was associated with higher scores on the Hospital Anxiety and Depression Scale (HADS) questionnaire in all four studies identified [18, 20, 30, 38, 41]. Similarly, three studies identified an association between FCR and worse outcomes in quality of life scales such as the functional assessment of cancer therapy (FACT-P) and short form 12 (SF 12) [18, 38, 39]. Lastly, Van de Wal et al. found a positive association between FCR and urinary, bowel and hormonal symptoms alongside distress scores [18].

**Table 3:** Predictors and consequences of FCR and PSA anxiety

Associated factor or outcome	FCR			PSA anxiety		
	( +) association	( −) association	(Nil) association	( +) association	( −) association	(Nil) association
Patient demographics						
Age	[46]c	[36]d [30],b, [38]b, [20]b, [25]a [18],a	[24]b, [41]b		[36]d, [11]d	[30]b [20],b, [38]b, [41]b
Education		[30]b, [46]c	[18]a, [41]b		[41]b	[30]b
Employment			[36]d			[36]d
Any religious belief			[36]d			[36]d
Had children			[36]d [18],a			[36]d
Cancer and treatment						
Time since treatment		[37]c	[18]a		[44]d	
Stage	[30]b		[36]d, [24]b, [25]a			[36]d, [30]b
RT			[36]d			[36]d
Latest PSA Value	[30, 38]b, [18]a		[30]b, [24]b, [25]a	[44]d, [11]d		[30]b
Physical symptoms						
Hormonal	[18]a		[36]d	[36]d		
Bowel	[18]a		[36]d	[36]d		
Urinary	[18]a, [25]a		[36]d	[36]d		
Sexual problems	[38]b, [30]b, [18]a		[36]d [18],a, [25]a			[36]d
Medical history						
Family history of Prostate cancer			[18]a, [24]b, [46]c	[44]d, [11]d		
Psychological						
Mental HRQOL		[37]c, [39]c				
Depression			[46]c	[44]d, [11]d		
Anxiety	[24]b, [46]c			[44]d, [11]d		
Distress	[18]a, [59]c					
Avoidance/intrusion	[18]a, [39]c					
HADS total score	[30]b, [20]b, [38]b [18],a, [41]b			[30]b, [38]b [20],b, [41]b		
STAI	[38]b					[38]b
FCR				[38]b, [30]b, [18]a		
Emotional	[27]b			[27]b		
Wellbeing and QOL measures						
Global QOL		[18]a, [41]b, [59]c			[29]d, [41]b	
Relationship satisfaction			[36]d		[36]d	
IES	[18]a					
FACT-P		[38]b, [39]c				[38]b
SF12		[20]b			[20]b	

Association evaluation: a, mean comparison; b, correlations; c, univariate regression models analysis; d, multivariate regression models analysis; e, modelling analysis

Index: *FACT-P*, functional assessment of cancer therapy prostate; *FCR*, fear of cancer recurrence; *HRQOL*, health-related quality of life; *HADS*, Hospital Anxiety and Depression Scale, *HRQOL*, health-related quality of life; *IES*, impact of event scale; *PSA*, prostate-specific antigen; *QOL*, quality of life; *RT*, radiation therapy; *SF12*, short form 12; *STAI*, state trait anxiety inventory

### PSA anxiety

#### Prevalence

Four studies measured prevalence of significant PSA anxiety ranging from 1.2 to 27.9% (median = 22.75) totalling 5416 patients [11, 38, 39, 43]. Anderson et al. [38] found a prevalence of 1.2% of significant PSA anxiety for patients undergoing active surveillance, in comparison to findings from Meissner et al. of 3% in radical prostatectomy patients in their large cohort of 4719 patients [11]. Higher prevalences were seen in other studies of 27% and 15% for those receiving hormone treatment or radiotherapy respectively [43]. Only one low risk of bias studies measured PSA anxiety prevalence finding 3% [11]. This was compared to a median prevalence of 20.2 for moderate risk of bias studies [38, 39, 43].

#### Severity and progression over time

Like FCR, the MAXPC PSA subscale was the most used measure to measure PSA anxiety severity with 16 of the 18 studies using it, with a total of three measures used overall. A cut-off of 4.5 is considered significant for this subscale. Overall scores were low in studies. At diagnosis, five studies measured PSA anxiety with the MAXPC PSA [19, 22, 36, 39, 44] with scores ranging from 0.25 to 2.43 (median = 0.79, IQR = 1.20). Four studies measured PSA anxiety between 6 and 10 months with scores of between 0.3 and 1.27 [17, 27, 36, 39] and eight studies at over a year post diagnosis (range = 0.27–2.08, median = 0.54, IQR = 0.85) with ongoing = low scores event at up to 10 years follow-up. Throughout the survivorship trajectory, no studies identified mean scores above the cut-off. Trends across longitudinal studies showed a low peak PSA anxiety at diagnosis, remaining low at up to 3 years after [17, 19, 22, 36].

#### Predictors of PSA anxiety

Due to the nature of active surveillance, more studies specifically looked at this population [17, 19, 22, 38, 39] which stayed below significance after a year when evaluated longitudinally [17, 22]. Overall, few studies compared between cancer characteristics and different treatments. However, where evaluated, no association was observed between cancer stage and PSA anxiety [30, 36]. Similar to FCR, gay and bisexual patients were found to have higher levels than heterosexual patients [19]. The relationship with age and PSA anxiety demonstrated mixed findings. Four studies identified nil association [20, 30, 38, 41]; however, two [36, 44] reported a negative association suggesting younger age to be associated with PSA anxiety.

#### Relationship to other outcomes

The strongest correlation between PSA anxiety and any other outcome was to overall HADS scores. All three studies identified [20, 30, 38] found a positive association between these scores, implying an association between PSA anxiety and wider mental health conditions such as depression and generalised anxiety. Furthermore, Anderson et al. [38] found a positive association between PSA anxiety and two other measures: FACT-P and the state trait anxiety inventory (STAI), further highlighting the association with generalised anxiety and suffering with other prostate-related symptoms. Interestingly, a close positive association between FCR and PSA anxiety was found in all three studies evaluating this [18, 30, 38]. These two mental wellbeing issues therefore seem to be closely interlinked, with patients possessing higher FCR having higher PSA anxiety [18]. Lastly, one study found a negative association between bowel, hormonal, urinary symptoms and PSA anxiety, although this correlation was small (*β* < 0.1) [36].

### Risk of bias assessment

Overall, studies demonstrated good internal and external validity. JBI checklist scores ranged from 4 to 9 (median = 6, IQR = 3). Eight studies demonstrated a low risk of bias, 24 a moderate risk of bias and six a high risk of bias and were excluded from final inclusion (online resource 5 and 6). Common concerns identified included high dropout rates of participants in cohort studies (*n* = 7), a lack of strategies to address these dropouts (*n* = 17) and no consideration of confounders in study design (*n* = 22). Furthermore, many studies demonstrated a poor wider representation of the prostate cancer population, focussing only on certain demographics or introduced selection bias through focussed electronic recruitment on social media or through email dispersal.

## Discussion

This review provides an overview of the current evidence for FCR and PSA anxiety in prostate cancer. We identified significant prevalences of FCR, and severity was moderate to low throughout survivorship with little variability over time. These findings are consistent with previous reviews evaluating all cancer patients, where levels of FCR were moderate to low, and importantly FCR is moderate at diagnosis and years after treatment [5]. However, findings were slightly lower than in testicular cancer, but with the younger age at diagnosis of this cohort this may be expected [47]. Our identified strongest predictors of FCR also corresponded to previous cancer literature, with younger age and physical symptoms experienced important factors [48]. This matches the understood perception of FCR being highly dependent on triggers — such as scans and appointments — and unless results are recorded at this trigger, scores may not reflect actual levels of FCR [48]. However, unlike previous cancer reviews [49], we did identify an association between advanced stage of disease and FCR within prostate cancer. Furthermore, evidence suggests that high levels of FCR correlate with poorer scores on other mental health and quality of life measures [50]. This matches what is known regarding anxiety and distress in prostate cancer patients with younger age and advanced stage of disease identified as significant risk factors [51, 52]. This suggests that FCR is linked to anxiety and depression. However, this review did not similarly identify smoking status, alcohol intake and comorbid health conditions as a predictor of FCR.

Surprisingly, whilst PSA anxiety prevalence was moderate, severity scores were overall low. This is however in keeping with previous evidence demonstrating PSA testing had little impact on anxiety or depression scores [53]. Similarly, for active surveillance, where clinicians are worried about patient PSA anxiety, cancer-specific anxiety levels have previously been reported as remaining low [48]. As expected, PSA anxiety seems to peak on entrance to active surveillance and decrease thereafter; however, most appear to already start with low levels. In addition, PSA anxiety when experienced is associated with other mental health symptoms and is inherently linked to FCR. Unsurprisingly, these two wellbeing measures are significantly associated meaning if patients experience one, they are likely to be experiencing the other. The triggers of each construct are complex with time since diagnosis levels not always decreasing as patients progress along their cancer journey, with [46] finding high levels many years post diagnosis. Potentially, they are related to the presence of symptoms [18]; however, high levels around specific appointments could act as a trigger to increased levels of FCR and PSA anxiety and should be further explored in future research.

There are important limitations of the currently available literature. Findings are reliable on observational studies limited by potential confounders, meaning the associations identified cannot be attributed to absolute causality. As an example, FCR in survivors can affect their behaviours with decreased exercise activity and association with increased alcohol consumption therefore possibly affecting physical outcomes [54]. Furthermore, the directionality of these relationships is difficult to assess. Due to the complex relationship between mind, body and environmental factors, it is hard to ascertain whether factors such as depression, anxiety, physical symptoms or relationship problems are associated with higher likelihood of significant FCR and PSA anxiety or whether the reverse relationship is true [29]. Additionally, there was a particular lack of comparative data between treatment groups and different populations, to inform clinicians of who is most likely to suffer with symptoms. Importantly, coping styles, psychological traits and previous mental health illness have not been assessed as predictors of FCR and PSA anxiety and considering the predictive role they have in other cancers for worse mental health [55], they should be specific factors that are considered in future research. Lastly, few longitudinal studies with regular timepoint intervals of measurement were conducted meaning progression over time is at times difficult to definitively establish.

Future research should aim to address these limitations. Further longitudinal studies with regular timepoints are required to better understand trajectories of FCR or PSA anxiety. Additionally, it has previously been demonstrated that different states of PSA levels such as increasing, stable and decreasing affected prostate cancer–specific anxiety, demonstrating the need for measuring timepoints around PSA blood tests and at more regular intervals. More work also needs to be conducted to identify factors associated with increased levels of FCR or PSA anxiety with differences between treatment groups of particular importance. Specifically, identifying differences between patients undergoing radical treatment versus active surveillance is of importance, better enabling clinicians and patients to make informed treatment choices. Similarly, further patient factors such as race need further investigation with research so far focussing only on the validity of psychometric tools in Black populations, with little further exploration. As identified previously, FCR is linked to anxiety and depression in prostate cancer and it should be evaluated whether they have similar predictors such as comorbid health conditions, income, alcohol intake and smoking status.

### Clinical implications

Clinically this review has important implications. Firstly, these issues have been demonstrated to be important during routine care in view of their prevalence and due to their association with other quality of life outcomes including physical and psychological wellbeing measures. Currently, the European Association of Urology [56] American association of urology [57] acknowledges that quality of life in prostate cancer can be reduced; however, it does not mention FCR as an unmet need in patients. Furthermore, whilst they acknowledge the impact of these mental wellbeing issues, the method of assessment and the treatment for these is not incorporated into the guidelines. Men with prostate cancer have specific and complex unmet needs and there is a need for a greater men-centred approach to address these issues, which include previously known issues of altered body image and masculinity as a result of altered sexual function, and also FCR and PSA anxiety as identified in this review.

This review however demonstrates that these domains should be considered important elements of quality of life post diagnosis and as such require further attention during the patient journey. We identify that anxiety levels appear to be highest at diagnosis giving a strong focussing point for their evaluation. Furthermore, some of the associated features of these symptoms including younger age and advanced disease offer target groups who require the greatest attention. This is important as once symptoms are detected, referral to appropriate services should be made, with cognitive behavioural therapy a demonstrated low-cost intervention minimising FCR and should be included in prostate cancer guidelines [58].

### Study limitations

This review is not without limitations. As mentioned before, the data currently available is of high heterogeneity. We attempted to somewhat mitigate this by only including studies with validated tools. This heterogeneity in results, characteristics and outcomes meant a statistical pooling of results via a meta-analysis was not feasible. Additionally, due to the inclusion of only observational studies, whilst we were able to evaluate associations between FCR and PSA anxiety with other outcomes and factors, we are unable to definitively label these as causative factors, particularly when considering the complex relationship that likely exists between them. Lastly, with variable sources and only studies in the English language included, it is possible some potentially relevant articles were missed. We however tried to minimise this risk through our comprehensive search using multiple databases.

## Conclusions

PSA anxiety and FCR are prevalent symptoms experienced by prostate cancer patients with severity scores at moderate to low levels. Both symptoms appear to peak at initial diagnosis and treatment, with only a minimal gradual decrease afterwards. Few definitive associative factors have been identified for either symptom, with only younger age psychological symptoms or experiencing physical symptoms demonstrating some evidence of correlation. Clinicians should be aware of these issues as when experienced, can have a profound impact on mental health and quality of life for patients. This highlights the importance of these symptoms for the quality of life of patients and the greater need to consider them during follow-up care. Improved identification of symptoms through screening of high-risk individuals is required with early referral for appropriate and effective treatment once identified. Further evidence is required, focussing on direct comparison of treatment groups or patient factors to identify those at greater risk of developing these symptoms.

## Supplementary Information

Below is the link to the electronic supplementary material.

Supplementary file1 (DOCX 21 KB)

Supplementary file2 (DOCX 148 KB)

Supplementary file3 (DOCX 57 KB)

Supplementary file4 (DOCX 16 KB)

Supplementary file5 (DOCX 38 KB)

Supplementary file6 (DOCX 42 KB)

## Data Availability

Original data utilised for the analysis of this review is available bona fide researchers following reasonable requests to the corresponding author.
