# Supplementary material for: Fear of cancer recurrence and PSA anxiety in patients with prostate cancer: a systematic review
Source: Support Care Cancer. 2022 Feb 1;30(7):5577–89. doi: 10.1007/s00520-022-06876-z (PMC9135793; doi:10.1007/s00520-022-06876-z)
Supplement: Supplementary file 1 — Supplementary file1 (DOCX 21 KB) [file 520_2022_6876_MOESM1_ESM.docx]

**Online resource**

**Online resource 1: Full search strategy**

Medline (via Pubmed) search terms

Terms used were a mixture of Mesh Terms and relevant abbreviations. The search was conducted up to the 25/08/21 with no limit on start date for the databases. The following search terms were used and based upon the aims of the review:

("prostate cancer" OR "prostate neoplasms") AND ("FCR" OR "FoR" OR "Fear of Recurrence" OR Fear of Cancer Recurrence" OR "PSA anxiety" OR "PSA distress" OR "FoP" OR "Fear of progression" OR "Fear of Cancer progression OR "Cancer specific worry" OR "Cancer related worry" OR "cancer specific distress" OR "cancer related distress" OR "cancer specific anxiety" OR "cancer related anxiety" OR "prostate cancer specific worry" OR "prostate cancer related worry" OR "prostate cancer related anxiety" OR "prostate cancer specific anxiety" OR "prostate cancer related distress" OR "prostate cancer specific distress" OR "disease specific anxiety" OR "disease related anxiety" OR "disease specific worry" OR "disease related worry" OR "disease specific distress" OR "disease related distress")

Embase and PsychINFO search terms (via Ovidsp):

("prostate cancer" or "prostate neoplasms").mp. and ("FCR" or "FoR" or "Fear of Recurrence" or "Fear of Cancer Recurrence OR PSA anxiety OR PSA distress OR FoP OR Fear of progression OR Fear of Cancer progression" or "Cancer specific worry" or "Cancer related worry" or "cancer specific distress" or "cancer related distress" or "cancer specific anxiety" or "cancer related anxiety" or "prostate cancer specific worry" or "prostate cancer related worry" or "prostate cancer related anxiety" or "prostate cancer specific anxiety" or "prostate cancer related distress" or "prostate cancer specific distress" or "disease specific anxiety" or "disease related anxiety" or "disease specific worry" or "disease related worry" or "disease specific distress" or "disease related distress").ab,kw,mh,ti. [mp=ti, ab, hw, tn, ot, dm, mf, dv, kw, fx, dq, tc, id, tm, mh]

ISCRTN Registry terms:

Prostate cancer AND ((fear of recurrence) OR (FCR) OR (FoR) OR (Fear of cancer recurrence) OR (PSA anxiety))-

Number of Results

- Medline: 572
- Embase::502
- PyschInfo: 55
- ISCRTN Registry: 0 relevant studies identified out of 186 results.

Total number of results exported into reference manager- Rayyan QCRI: 1129 results

Potentially relevant studies identified from other sources (reference reviews): 2

After de duplication- 1148 records screened after de-duplication (962 from databases and 186 from registers)
