# Supplementary material for: Fear of cancer recurrence and PSA anxiety in patients with prostate cancer: a systematic review
Source: Support Care Cancer. 2022 Feb 1;30(7):5577–89. doi: 10.1007/s00520-022-06876-z (PMC9135793; doi:10.1007/s00520-022-06876-z)
Supplement: Supplementary file 2 — Supplementary file2 (DOCX 148 KB) [file 520_2022_6876_MOESM2_ESM.docx]

**Online resource 2: Full texts Excluded with reason.**

| Study | Title | Reason For exclusion |
| --- | --- | --- |
| Addington et al 2016 [3] | Can posttraumatic growth protect the mental and physical health of prostate cancer survivors? | Non- validated Measure |
| Admiraal et al 2013 [4] | Do cancer and treatment type affect distress? | Irrelevant outcome measure |
| Anderson et al 2013 [1] | Prostate specific antigen utility and anxiety in the management of prostate cancer by active surveillance | Conference abstract- no data |
| Anderson et al 2013 [6] | Anxiety in men with prostate cancer treated by active surveillance | Same Data set |
| Armes et al 2009 [7] | Patients' supportive care needs beyond the end of cancer treatment: A prospective, longitudinal survey | No PC specific data |
| Baider et al 2003 [8] | Is perceived family support a relevant variable in psychological distress?. A sample of prostate and breast cancer couples | Irrelevant outcome measure |
| Bailey et al 2014[9] | Psychosocial trajectories of men monitoring prostate-specific antigen levels following surgery for prostate cancer | Full text unavailable |
| Batista-Miranda et al 2003 [10] | Quality of life in prostate cancer patients and controls: psychometric validation of the FACTP-4 in Spanish, and relation to urinary symptoms | Full text unavailable |
| Batty et al 2017 [11] | Psychological distress in relation to site specific cancer mortality: pooling of unpublished data from 16 prospective cohort studies | Irrelevant outcome measure |
| Befort et al 2005 [12] | A measure of health-related quality of life among patients with localized prostate cancer: results from ongoing scale development | Non- validated measure |
| Bellardita et al 2013[14] | Predictors of health-related quality of life and adjustment to prostate cancer during active surveillance | Conference abstract |
| Bellardita et al 2015 [13] | How does active surveillance for prostate cancer affect quality of life? A systematic review | Review |
| Ben-Tovim et al 2002[15] | Coping with prostate cancer: a quantitative analysis using a new instrument, the centre for clinical excellence in urological research coping with cancer instrument | Irrelevant outcome measure |
| Bergman et al 2009 [16] | Partnership and outcomes in men with prostate cancer | Irrelevant outcome measure |
| Bisson et al 2002 [18] | The prevalence and predictors of psychological distress in patients with early localized prostate cancer | Irrelevant outcome measure |
| Bill-Axelson et al 2013 [17] | Long-term distress after radical prostatectomy versus watchful waiting in prostate cancer: a longitudinal study from the Scandinavian Prostate Cancer Group-4 randomized clinical trial | Irrelevant outcome measure |
| Burke et al 2003 [19] | Emotional and cognitive burden of prostate cancer | Irrelevant outcome measure |
| Burney S et al | Regular PSA testing anxiety for disease management and PSA utility in men with prostate cance | Full text unavailable |
| Buzaglo et al 2017 [20] | Sexual function, quality of life, and cancer-related distress among prostate cancer survivors | Irrelevant outcome measure |
| Castermans et al 2016 [21] | Psychosocial screening for patients with prostate cancer: The development and validation of the psychosocial distress questionnaire-prostate cancer | Irrelevant outcome measure |
| Chambers et al 2013 [22] | Systematic review of research into the psychological aspects of prostate cancer in Asia: what do we know? | Review |
| Chatre et al 2011 [23] | Racial differences in well-being and cancer concerns in prostate cancer patients | Irrelevant outcome measure |
| Chien et al 2016 [2] | Effects of appraisals of ilness on anxiety and quality of life in couples of prostate cancer | Duplicate data set |
| Chien et al 2019 [24] | Prostate cancer-specific anxiety and the resulting health-related quality of life in couples | Duplicate data set |
| Chou et al 2020 [25] | The Psychometric Properties of Taiwanese Version of the Memorial Anxiety Scale for Prostate Cancer | Duplicate data set |
| Clark et al 2003 [26] | Patients' perceptions of quality of life after treatment for early prostate cancer | Irrelevant outcome measure |
| Cliff et al 2000 [27] | Psychosocial morbidity in prostate cancer: II. A comparison of patients and partners | Irrelevant outcome measure |
| Colloca et al 2016[28] | The Effects of Social Support on Health-Related Quality of Life of Patients with Metastatic Prostate Cance | Review |
| Couper et al 2010 [29] | Predictors of psychosocial distress 12 months after diagnosis with early and advanced prostate cancer. | Irrelevant outcome measure |
| Custers et al 2018 [30] | Re-validation and screening capacity of the 6-item version of the Cancer Worry Scale | No PC specific data |
| Dale et al 2007 [32] | Extending the validity of the Memorial Anxiety Scale for Prostate Cancer (MAX-PC) at the time of prostate biopsy in a racially-mixed population | Irrelevant outcome measure |
| Dall'Era 2015 [33] | Patient and disease factors affecting the choice and adherence to active surveillance | Review |
| da silva 1993 [31] | Quality of life in prostatic cancer patients | Irrelevant outcome measure |
| Davison 2003[34] | Decisional regret and quality of life after participating in medical decision-making for early-stage prostate cancer | Irrelevant outcome measure |
| Deimling et al 2017 [36] | Personality and psychological distress among older adult, long-term cancer survivors | Duplicate data set |
| Deimling et al 2017 [37] | The relative importance of cancer-related and general health worries and distress among older adult, long-term cancer survivors | Duplicate data set |
| De Luca et al [35] | Cancer specific anxiety and depression levels in localized low-risk prostate cancer who choose active surveillance or radical treatment: Findings from the START study | Conference abstract- no data |
| Diefenbach et al 2008 [38] | Longitudinal associations among quality of life and its predictors in patients treated for prostate cancer: the moderating role of age | Non- validated Measure |
| Dinkel et al 2014 [39] | Influence of family history on psychosocial distress and perceived need for treatment in prostate cancer survivors | Irrelevant outcome measure |
| Dordoni et al 2020 [97] | The relationship between prostate cancer anxiety and mindfulness ability in patients with PCA | Irrelevant outcome measure |
| Dordoni et al 2017 [5] | Health-related quality of life of men on active surveillance: Association with psychological factors | Irrelevant outcome measure |
| Dräger et al 2017 [40] | Psychosocial stress in patients with prostate cancer : Experiences by using psychooncological screening questionnaires | Non- english language |
| Drummond et al 2015 [41] | Establishing a population-based patient-reported outcomes study (PROMs) using national cancer registries across two jurisdictions: the Prostate Cancer Treatment, your experience (PiCTure) study | Irrelevant outcome measure |
| Du et al 1997 [42] | Study on the quality of life in patients with prostate cancer | Non- English language |
| Egger et al 2018 [43] | Factors associated with the use of complementary and alternative medicines for prostate cancer by long-term survivors | Duplicate data set |
| Erim et al 2020 [44] | Associations between prostate cancer-related anxiety and health-related quality of life | Irrelevant outcome measure |
| Fosså 2016 [46] | Reduction of quality of life in prostate cancer patients: experience among 6200 men in the Nordic countries | Non validated measure |
| Fowler et al 1996 [47] | Outcomes of external-beam radiation therapy for prostate cancer: a study of Medicare beneficiaries in three surveillance, epidemiology, and end results areas | Irrelevant outcome measure |
| Frydenberg et al | Psychological stress associated with active surveillance for localised low risk prostate cancer | Full text unavailable |
| Germino et al 1998 [48] | Uncertainty in prostate cancer. Ethnic and family patterns | Irrelevant outcome measure |
| Giese-Davis 2012 [49] et al | Screening for distress, the 6th vital sign: Common problems in cancer outpatients over one year in usual care: Associations with marital status, sex, and age | Irrelevant outcome measure |
| Giesler JM; Weis J 2021 [50] | Patient competence in the context of cancer: its dimensions and their relationships with coping, coping self-efficacy, fear of progression, and depression | Irrelevant outcome measure |
| Glaser et al 2013 [51] | Patient-reported outcomes of cancer survivors in England 1-5 years after diagnosis: A cross-sectional survey | No PC specific data |
| Goltz H | Fear of recurrence among low-health literacy veterans with prostate cancer | Full text unavailable |
| Gore et al 2010 [52] | Correlates of bother following treatment for clinically localized prostate cancer | Irrelevant outcome measure |
| Green et al 2002 [53] | Coping and health-related quality of life in men with prostate cancer randomly assigned to hormonal medication or close monitoring | Irrelevant outcome measure |
| Groarke et al 2020 [54] | Quality of life and adjustment in men with prostate cancer: Interplay of stress, threat and resilience | Irrelevant outcome measure |
| Guan et al 2020 [55] | Illness uncertainty, coping, and quality of life among patients with prostate cancer | Irrelevant outcome measure |
| Hack et al 2010 [56] | Predictors of distress and quality of life in patients undergoing cancer therapy: impact of treatment type and decisional role | Irrelevant outcome measure |
| Hamilton et al 2016 [57] | The perspective of prostate cancer patients and patients' partners on the psychological burden of androgen deprivation and the dyadic adjustment of prostate cancer couples | Irrelevant outcome measure |
| Harden et al [58] | The influence of developmental life stage on quality of life in survivors of prostate cancer and their partners | Irrelevant outcome measure |
| Hart 2008 [59] et al | Fear of recurrence, treatment satisfaction, and quality of life after radical prostatectomy for prostate cancer | Duplicate data set |
| Heo et al 2020 [60] | Psychological distress among prostate cancer survivors in South Korea: A nationwide population-based, longitudinal study. | Irrelevant outcome measure |
| Herkommer et al 2011 [61] | Psychosocial distress in prostate cancer patients: Does family history make a difference? | Irrelevant outcome measure |
| Herr et al 2000 [62] | Quality of life of asymptomatic men with nonmetastatic prostate cancer on androgen deprivation therapy | Irrelevant outcome measure |
| Hong et al 2010 [64] | Impact of radical prostatectomy positive surgical margins on fear of cancer recurrence: results from CaPSURE | Duplicate data set |
| Isaka [66] | [QOL assessment in the treatment of prostate cancer] | Non- English language |
| Ilie et al 2020 [65] | Current Mental Distress Among Men With a History of Radical Prostatectomy and Related Adverse Correlates | Irrelevant outcome measure |
| Jenkins et al 2004 [67] | Sexuality and health-related quality of life after prostate cancer in African-American and white men treated for localized disease | Irrelevant outcome measure |
| Jones et al 2017 [68] | Association of worry about cancer to benefit finding and functioning in long-term cancer survivors | High risk of bias |
| Joshy et al 2020 [69] | Disability, psychological distress and quality of life in relation to cancer diagnosis and cancer type: population-based Australian study of 22,505 cancer survivors and 244,000 people without cancer | Irrelevant outcome measure |
| Kim et al 2017 [70] | Predictors of health-related quality of life in Korean prostate cancer patients receiving androgen deprivation therapy | Irrelevant outcome measure |
| Kim et al 2008 [71] | Quality of life of couples dealing with cancer: dyadic and individual adjustment among breast and prostate cancer survivors and their spousal caregivers | Irrelevant outcome measure |
| Kirschner-Hermanns et Jakse 2002 [72] | Quality of life following radical prostatectomy | Review |
| Klotz 2013 [73] | Active surveillance, quality of life, and cancer-related anxiety | Opinion |
| Knapp et al 2012 [74] | Trajectories and predictors of symptom occurrence, severity, and distress in prostate cancer patients undergoing radiation therapy. | Irrelevant outcome measure |
| Koch et al 2013 [75] | Fear of recurrence and disease progression in long-term cancer survivors | Review |
| Kornblith et al 1994 [76] | Quality of life of patients with prostate cancer and their spouses. The value of a data base in clinical care | Irrelevant outcome measure |
| Kypriotakis et al 2016 [77] | Correlated and Coupled Trajectories of Cancer-Related Worries and Depressive Symptoms among Long-Term Cancer Survivors | No PC specific data |
| Latini et al 2007 [78] | The relationship between anxiety and time to treatment for patients with prostate cancer on surveillance | Duplicate data set |
| Lebel et al 2013 [80] | Does fear of cancer recurrence predict cancer survivors' health care use | No PC specific data |
| Lebel et al 2016 [79] | Empirical validation of the English version of the Fear of Cancer Recurrence Inventory | High risk of bias |
| Leclair et al [108] | The Relationship between Fear of Cancer Recurrence and Health Behaviors: A Nationwide Longitudinal Study of Cancer Survivors | No PC specific data |
| Lilleby et al 1999 [81] | Long-term morbidity and quality of life in patients with localized prostate cancer undergoing definitive radiotherapy or radical prostatectomy | Irrelevant outcome measure |
| Loeb et al 2018 [82] | Health state utilities among contemporary prostate cancer patients on active surveillance | Irrelevant outcome measure |
| Lubeck et al 2001 [83] | Health related quality of life differences between black and white men with prostate cancer: data from the cancer of the prostate strategic urologic research endeavor. | Irrelevant outcome measure |
| Maggi et al 2019 [84] | Psychological impact of different primary treatments for prostate cancer: A critical analysis | Review |
| Maguire et al [85] | Regret and fear in prostate cancer: The relationship between treatment appraisals and fear of recurrence in prostate cancer survivors | High risk of bias |
| McGinty et al 2015 [86] | Coping style mediates the relationship between symptom burden and cancer specific distress in men with advanced prostate cancer | Full text unavailable |
| Mehnert et al 2008 [87] | Psychometric evaluation of the German version of the Life Attitude Profile-Revised (LAP-R) in prostate cancer patients | Irrelevant outcome measure |
| Mehnert et al 2010 [88] | Depression, anxiety, post-traumatic stress disorder and health-related quality of life and its association with social support in ambulatory prostate cancer patients. | Irrelevant outcome measure |
| Mellon et al[89] | A family-based model to predict fear of recurrence for cancer survivors and their caregivers | No PC specific data |
| Meropol et al [90] | Cancer patient preferences for quality and length of life | Irrelevant outcome measure |
| Müller G; Otto U [91] | [Quality of life, psychological distress, and social outcomes after radical prostatectomy. Results from a urology competence center for rehabilitation] | Non-English Language |
| Nelson et al 2011 [92] | Validation of the memorial anxiety scale for prostate cancer in African-American men with prostate cancer | Same data set |
| Nelson et al 2009 [93] | The chronology of distress, anxiety, and depression in older prostate cancer patients | Irrelevant outcome measure |
| Oba et al 2017 [94] | Psychological distress in men with prostate cancer and their partners before and after cancer diagnosis: a longitudinal study. | Irrelevant outcome measure |
| Occhipinti et al 2019 [95] | A prospective study of psychological distress after prostate cancer surgery. | Irrelevant outcome measure |
| Ottenbacher et al 2013 [96] | Cancer-specific concerns and physical activity among recently diagnosed breast and prostate cancer survivors. | Irrelevant outcome measure |
| Otto et al [63] | Don't google 'prostate cancer' - Information seeking behavior and disease specific anxiety among men with localized prostate cancer | Non-Validated measure |
| Pastore et al 2017 [99] | Psychological distress in patients undergoing surgery for urological cancer: A single centre cross-sectional study | Irrelevant outcome measure |
| Parker et al 2010 [98] | Pre-treatment PSA level and Gleason score are not associated with cancer-specific anxiety prior to surgical treatment for newly diagnosed prostate cancer | Irrelevant outcome measure |
| Perczek et al 2002 [100] | Facing a prostate cancer diagnosis: who is at risk for increased distress? | Irrelevant outcome measure |
| Philip et al [101] | Obesity and psychosocial well-being among cancer patients and survivors | Irrelevant outcome measure |
| Potosky et al 2001 [102] | Quality-of-life outcomes after primary androgen deprivation therapy: results from the Prostate Cancer Outcomes Study | Irrelevant outcome measure |
| Resnick et al [104] | Factors associated with satisfaction with prostate cancer care: Results from CaPSURE | Duplicate data set |
| Ream et al 2008 [103] | Supportive care needs of men living with prostate cancer in England: a survey. | Irrelevant outcome measure |
| Rönningås et al 2019 [106] | Prostate-specific antigen (PSA) and distress: - a cross-sectional nationwide survey in men with prostate cancer in Sweden. | Irrelevant outcome measure |
| Roeloffzen et al 2010 [105] | Health-Related Quality of Life up to Six Years After 125I Brachytherapy for Early-Stage Prostate Cancer | Irrelevant outcome measure |
| Ruane-McAteer et al 2016 [45] | We can cure, but can we care? Favourable-risk disease favourable psychological wellbeing in men recently diagnosed with PCa: Baseline findings from a prospective, longitudinal study | Conference abstract- no data |
| Schouten et al 2019 [107] | Systematic screening and assessment of psychosocial well-being and care needs of people with cancer | Review |
| Seiler et al 2012 [109] | Protocol-based active surveillance for low-risk prostate cancer: anxiety levels in both men and their partners | Irrelevant outcome measure |
| Sharp  et al 2016 [110] | Cancer-related symptoms predict psychological wellbeing among prostate cancer survivors: results from the PiCTure study | Irrelevant outcome measure |
| Smith et al 2015 [111] | Ten-year quality of life and psychological outcomes of men managed entirely with active surveillance: The NSW Prostate Cancer Care and Outcomes Study | No full text available |
| Smith et al [112] | Understanding Long-Term Cancer Survivors' Preferences for Ongoing Medical Care | No PC specific data |
| Soloway et al 2005 [113] | Sexual, psychological and dyadic qualities of the prostate cancer 'couple'. | Irrelevant outcome measure |
| Tan et al 2016 [114] | The Relationship between Intolerance of Uncertainty and Anxiety in Men on Active Surveillance for Prostate Cancer. | Irrelevant outcome measure |
| Torbit et al 2015 [115] | Fear of recurrence: The importance of self-efficacy and satisfaction with care in gay men with prostate cancer | Irrelevant outcome measure |
| Traeger et al 2009 [116] | Illness perceptions and emotional well-being in men treated for localized prostate cancer. | Irrelevant outcome measure |
| Turner et al 2009 [117] | Psychological distress and prostate specific antigen levels in men with and without prostate cancer | Irrelevant outcome measure |
| Tutino et al 2020 | Active surveillance vs active treatment: Psychological predictors | Conference abstract- no data |
| Ullrich et al 2003 [118] | Cancer fear and mood disturbance after radical prostatectomy: consequences of biochemical evidence of recurrence | Irrelevant outcome measure |
| Vasarainen et al 2012 [121] | Prostate cancer active surveillance and health-related quality of life: results of the Finnish arm of the prospective trial. | Irrelevant outcome measure |
| van den Bergh et al 2009 [119] | Prostate cancer-specific anxiety in Dutch patients on active surveillance: validation of the memorial anxiety scale for prostate cancer | Duplicate data set |
| Van stam et al 2020[120] | Patient-reported Outcomes Following Treatment of Localised Prostate Cancer and Their Association with Regret About Treatment Choices | High risk of bias |
| Venderbos et al 2019 [122] | Eight year patient reported outcome data of the first 150 Dutch men on active surveillance in the Prostate cancer Research International Active Surveillance study (PRIAS) | Duplicate Data set |
| Victorson et al 2016 [123] | Predictors of quality of life in men with localized prostate cancer: The role of treatment decision making satisfaction, neuroticism, and sexual functioning | High risk of bias |
| Visser et al 2003 [124] | Changes in health-related quality of life of men with prostate cancer 3 months after diagnosis: the role of psychosocial factors and comparisment with benign prostate hyperplasia patients | Irrelevant outcome measure |
| Wallerstedt et al 2019 [125] | Quality of Life After Open Radical Prostatectomy Compared with Robot-assisted Radical Prostatectomy. | Irrelevant outcome measure |
| Watson et al 2016 [126] | Symptoms, unmet needs, psychological well-being and health status in survivors of prostate cancer: implications for redesigning follow-up | Irrelevant outcome measure |
| Wilcox et al 2014 [128] | Quantifying anxiety and quality of life amongst patients on active surveillance for prostate cancer in an Australian setting | High risk of bias |
| White et al 2012 [127] | Mapping the psychosocial and practical support needs of cancer patients and their families in Western Australia | No PC specific data |
| Wilding et al 2019 [129] | Cancer-related symptoms, mental well-being, and psychological distress in men diagnosed with prostate cancer treated with androgen deprivation therapy | Irrelevant outcome measure |
| Wollersheim et al 2020 [130] | Design of the PROstate cancer follow-up care in Secondary and Primary hEalth Care study (PROSPEC): a randomized controlled trial to evaluate the effectiveness of primary care-based follow-up of localized prostate cancer survivors | Irrelevant outcome measure |
| Wu et al 2019 [131] | Longitudinal dyadic associations of fear of cancer recurrence and the impact of treatment in prostate cancer patients and their spouses | Non validated measure |
| Yanez et al 2015 [132] | The importance of perceived stress management skills for patients with prostate cancer in active surveillance. | Irrelevant outcome measure |
| Zajdlewicz et al 2017 [133] | Health-Related Quality of Life After the Diagnosis of Locally Advanced or Advanced Prostate Cancer: A Longitudinal Study. | Irrelevant outcome measure |

**References**

1. (2013) Abstracts Psycho-Oncology 22: 124-362

2. (2016) ICCN 2016 Abstract Book: Oral Sessions Cancer Nursing 39

3. Addington EL (2016) Can posttraumatic growth protect the mental and physical health of prostate cancer survivors?

4. Admiraal JM, Reyners AK, Hoekstra-Weebers JE (2013) Do cancer and treatment type affect distress? Psychooncology 22: 1766-1773

5. al De (2017) Health-related quality of life of men on active surveillance: Association with psychological factors, Abstracts of the 27th annual meeting of the italian society of uro-oncology (siuro) Anticancer Research 37: 2051

6. Anderson J, Riciardelli L, Burney S, Frydenberg M, Fletcher J, Brooker J (2013) Anxiety in men with prostate cancer treated by active surveillance BJU International 112: 5-5

7. Armes J, Crowe M, Colbourne L, Morgan H, Murrells T, Oakley C, Palmer N, Ream E, Young A, Richardson A (2009) Patients' supportive care needs beyond the end of cancer treatment: a prospective, longitudinal survey J Clin Oncol 27: 6172-6179

8. Baider L, Ever-Hadani P, Goldzweig G, Wygoda MR, Peretz T (2003) Is perceived family support a relevant variable in psychological distress?. A sample of prostate and breast cancer couples J Psychosom Res 55: 453-460

9. Bailey DE, Jr., Wallace Kazer M, Polascik TJ, Robertson C (2014) Psychosocial trajectories of men monitoring prostate-specific antigen levels following surgery for prostate cancer Oncol Nurs Forum 41: 361-368

10. Batista-Miranda JE, Sevilla-Cecilia C, Torrubia R, Musquera M, Huguet-Pérez J, Ponce de León X, Rosales Bordes A, Salvador Bayarri J, Villavicencio Mavrich H (2003) Quality of life in prostate cancer patients and controls: psychometric validation of the FACTP-4 in Spanish, and relation to urinary symptoms Arch Esp Urol 56: 447-454

11. Batty GD, Russ TC, Stamatakis E, Kivimäki M (2017) Psychological distress in relation to site specific cancer mortality: pooling of unpublished data from 16 prospective cohort studies Bmj 356: j108

12. Befort CA, Zelefsky MJ, Scardino PT, Borrayo E, Giesler RB, Kattan MW (2005) A measure of health-related quality of life among patients with localized prostate cancer: results from ongoing scale development Clin Prostate Cancer 4: 100-108

13. Bellardita L, Valdagni R, van den Bergh R, Randsdorp H, Repetto C, Venderbos LD, Lane JA, Korfage IJ (2015) How does active surveillance for prostate cancer affect quality of life? A systematic review Eur Urol 67: 637-645

14. Bellardita Lea (2013) Predictors of health-related quality of life and adjustment to prostate cancer during active surveillance, Abstracts of the 23rd annual meeting of the italian society of uro-oncology (siuro) Anticancer Research 33: 2245

15. Ben-Tovim DI, Dougherty ML, Stapleton AM, Pinnock CB (2002) Coping with prostate cancer: a quantitative analysis using a new instrument, the centre for clinical excellence in urological research coping with cancer instrument Urology 59: 383-388

16. Bergman J, Gore JL, Saigal CS, Kwan L, Litwin MS (2009) Partnership and outcomes in men with prostate cancer Cancer 115: 4688-4694

17. Bill-Axelson A, Garmo H, Holmberg L, Johansson JE, Adami HO, Steineck G, Johansson E, Rider JR (2013) Long-term distress after radical prostatectomy versus watchful waiting in prostate cancer: a longitudinal study from the Scandinavian Prostate Cancer Group-4 randomized clinical trial Eur Urol 64: 920-928

18. Bisson JI, Chubb HL, Bennett S, Mason M, Jones D, Kynaston H (2002) The prevalence and predictors of psychological distress in patients with early localized prostate cancer BJU Int 90: 56-61

19. Burke MA, Lowrance W, Perczek R (2003) Emotional and cognitive burden of prostate cancer Urol Clin North Am 30: 295-304

20. Buzaglo JS, Zaleta AK, Miller MF, Johnson J, Diefenbach MA, Lepore SJ, Nelson CJ, O'Rourke MA, Geynisman DM (2017) Sexual function, quality of life, and cancer-related distress among prostate cancer survivors Journal of Clinical Oncology 35: e16587-e16587

21. Castermans E, Coenders M, Beerlage HP, de Vries J (2016) Psychosocial screening for patients with prostate cancer: The development and validation of the psychosocial distress questionnaire-prostate cancer J Psychosoc Oncol 34: 512-529

22. Chambers SK, Hyde MK, Ip DF, Dunn JC, Gardiner RA (2013) Systematic review of research into the psychological aspects of prostate cancer in Asia: what do we know? Asian Pac J Cancer Prev 14: 2621-2626

23. Chhatre S, Wein AJ, Malkowicz SB, Jayadevappa R (2011) Racial differences in well-being and cancer concerns in prostate cancer patients J Cancer Surviv 5: 182-190

24. Chien C-H, Chuang C-K, Liu K-L, Pang S-T, Wu C-T, Chang Y-H (2019) Prostate cancer-specific anxiety and the resulting health-related quality of life in couples Journal of Advanced Nursing 75: 63-74

25. Chou MC, Chien CH, Chung HJ, Chuang CK, Wu CT, Pang ST, Liu KL, Chang YH (2020) The Psychometric Properties of Taiwanese Version of the Memorial Anxiety Scale for Prostate Cancer J Pain Symptom Manage

26. Clark JA, Inui TS, Silliman RA, Bokhour BG, Krasnow SH, Robinson RA, Spaulding M, Talcott JA (2003) Patients' perceptions of quality of life after treatment for early prostate cancer J Clin Oncol 21: 3777-3784

27. Cliff AM, MacDonagh RP (2000) Psychosocial morbidity in prostate cancer: II. A comparison of patients and partners BJU Int 86: 834-839

28. Colloca G, Colloca P (2016) The Effects of Social Support on Health-Related Quality of Life of Patients with Metastatic Prostate Cancer J Cancer Educ 31: 244-252

29. Couper JW, Love AW, Duchesne GM, Bloch S, Macvean M, Dunai JV, Scealy M, Costello A, Kissane DW (2010) Predictors of psychosocial distress 12 months after diagnosis with early and advanced prostate cancer Med J Aust 193: S58-61

30. Custers JAE, Kwakkenbos L, van de Wal M, Prins JB, Thewes B (2018) Re-validation and screening capacity of the 6-item version of the Cancer Worry Scale Psychooncology 27: 2609-2615

31. da Silva FC (1993) Quality of life in prostatic cancer patients Cancer 72: 3803-3806

32. Dale W, Hemmerich J, Meltzer D (2007) Extending the validity of the Memorial Anxiety Scale for Prostate Cancer (MAX-PC) at the time of prostate biopsy in a racially-mixed population Psychooncology 16: 493-498

33. Dall'Era MA (2015) Patient and disease factors affecting the choice and adherence to active surveillance Curr Opin Urol 25: 272-276

34. Davison BJ, Goldenberg SL (2003) Decisional regret and quality of life after participating in medical decision-making for early-stage prostate cancer BJU Int 91: 14-17

35. De Luca S, Bertetto O, Ciccone G, Rosato R, Galassi C, Bollito E, Gontero P, Garrou D, Cattaneo G, Amparore D, Checcucci E, Volpi G, Manfredi M, Fiori C, Porpiglia F (2019) SC46 - Cancer specific anxiety and depression levels in localized low-risk prostate cancer who choose active surveillance or radical treatment: Findings from the START study European Urology Supplements 18: e3184

36. Deimling GT, Albitz C, Monnin K, Renzhofer Pappada HT, Nalepa E, Boehm ML, Mitchell C (2017) Personality and psychological distress among older adult, long-term cancer survivors J Psychosoc Oncol 35: 17-31

37. Deimling GT, Brown SP, Albitz C, Burant CJ, Mallick N (2017) The relative importance of cancer-related and general health worries and distress among older adult, long-term cancer survivors Psycho-Oncology 26: 182-190

38. Diefenbach M, Mohamed NE, Horwitz E, Pollack A (2008) Longitudinal associations among quality of life and its predictors in patients treated for prostate cancer: the moderating role of age Psychol Health Med 13: 146-161

39. Dinkel A, Kornmayer M, Gschwend JE, Marten-Mittag B, Herschbach P, Herkommer K (2014) Influence of family history on psychosocial distress and perceived need for treatment in prostate cancer survivors Fam Cancer 13: 481-488

40. Dräger DL, Harke NN, Sievert KD, Protzel C, Hakenberg OW (2017) [Psychosocial stress in patients with prostate cancer : Experiences by using psychooncological screening questionnaires] Urologe A 56: 1445-1449

41. Drummond FJ, Kinnear H, Donnelly C, O'Leary E, O'Brien K, Burns RM, Gavin A, Sharp L (2015) Establishing a population-based patient-reported outcomes study (PROMs) using national cancer registries across two jurisdictions: the Prostate Cancer Treatment, your experience (PiCTure) study BMJ Open 5: e006851

42. Du SF, Shi LY, Zhang HJ (1997) [Study on the quality of life in patients with prostate cancer] Zhonghua Liu Xing Bing Xue Za Zhi 18: 95-97

43. Egger S, Hughes S, Smith DP, Chambers S, Kahn C, Moxey A, O'Connell DL (2018) Factors associated with the use of complementary and alternative medicines for prostate cancer by long-term survivors PloS one 13: e0193686-e0193686

44. Erim DO, Bennett AV, Gaynes BN, Basak RS, Usinger D, Chen RC (2020) Associations between prostate cancer-related anxiety and health-related quality of life Cancer Med 9: 4467-4473

45. et R-M (2016) We can cure, but can we care? Favourable-risk disease favourable psychological wellbeing in men recently diagnosed with PCa: Baseline findings from a prospective, longitudinal study, Abstracts Psycho-Oncology 25: 3-195

46. Fosså SD, Bengtsson T, Borre M, Ahlgren G, Rannikko A, Dahl AA (2016) Reduction of quality of life in prostate cancer patients: experience among 6200 men in the Nordic countries Scand J Urol 50: 330-337

47. Fowler FJ, Jr., Barry MJ, Lu-Yao G, Wasson JH, Bin L (1996) Outcomes of external-beam radiation therapy for prostate cancer: a study of Medicare beneficiaries in three surveillance, epidemiology, and end results areas J Clin Oncol 14: 2258-2265

48. Germino BB, Mishel MH, Belyea M, Harris L, Ware A, Mohler J (1998) Uncertainty in prostate cancer. Ethnic and family patterns Cancer Pract 6: 107-113

49. Giese-Davis J, Waller A, Carlson LE, Groff S, Zhong L, Neri E, Bachor SM, Adamyk-Simpson J, Rancourt KMS, Dunlop B, Bultz BD (2012) Screening for distress, the 6th vital sign: common problems in cancer outpatients over one year in usual care: associations with marital status, sex, and age BMC Cancer 12: 441

50. Giesler JM, Weis J (2021) Patient competence in the context of cancer: its dimensions and their relationships with coping, coping self-efficacy, fear of progression, and depression Support Care Cancer 29: 2133-2143

51. Glaser AW, Fraser LK, Corner J, Feltbower R, Morris EJA, Hartwell G, Richards M (2013) Patient-reported outcomes of cancer survivors in England 1–5 years after diagnosis: a cross-sectional survey BMJ Open 3: e002317

52. Gore JL, Gollapudi K, Bergman J, Kwan L, Krupski TL, Litwin MS (2010) Correlates of bother following treatment for clinically localized prostate cancer J Urol 184: 1309-1315

53. Green HJ, Pakenham KI, Headley BC, Gardiner RA (2002) Coping and health-related quality of life in men with prostate cancer randomly assigned to hormonal medication or close monitoring Psychooncology 11: 401-414

54. Groarke A, Curtis R, Skelton J, Groarke JM (2020) Quality of life and adjustment in men with prostate cancer: Interplay of stress, threat and resilience PLoS One 15: e0239469

55. Guan T, Santacroce SJ, Chen DG, Song L (2020) Illness uncertainty, coping, and quality of life among patients with prostate cancer Psychooncology 29: 1019-1025

56. Hack TF, Pickles T, Ruether JD, Weir L, Bultz BD, Mackey J, Degner LF (2010) Predictors of distress and quality of life in patients undergoing cancer therapy: impact of treatment type and decisional role Psychooncology 19: 606-616

57. Hamilton LD, Van Dam D, Wassersug RJ (2016) The perspective of prostate cancer patients and patients' partners on the psychological burden of androgen deprivation and the dyadic adjustment of prostate cancer couples Psychooncology 25: 823-831

58. Harden J, Northouse L, Cimprich B, Pohl JM, Liang J, Kershaw T (2008) The influence of developmental life stage on quality of life in survivors of prostate cancer and their partners J Cancer Surviv 2: 84-94

59. Hart SL, Latini DM, Cowan JE, Carroll PR (2008) Fear of recurrence, treatment satisfaction, and quality of life after radical prostatectomy for prostate cancer Support Care Cancer 16: 161-169

60. Heo J, Noh OK, Chun M, Oh YT, Kim L (2020) Psychological distress among prostate cancer survivors in South Korea: A nationwide population-based, longitudinal study Asia Pac J Clin Oncol 16: e125-e130

61. Herkommer K, Dinkel A, Kornmayer M, Herschbach P, Gschwend J (2011) 421 psychosocial distress in prostate cancer patients: Does family history make a difference? European Urology Supplements - eur urol suppl 10: 146-146

62. Herr HW, O'Sullivan M (2000) Quality of life of asymptomatic men with nonmetastatic prostate cancer on androgen deprivation therapy J Urol 163: 1743-1746

63. Hilger C, Otto I, Hill C, Huber T, Kendel F (2019) ["Dr. Google"-information-seeking behavior and disease-specific anxiety among men with localized prostate cancer] Urologe A 58: 1050-1056

64. Hong YM, Hu JC, Paciorek AT, Knight SJ, Carroll PR (2010) Impact of radical prostatectomy positive surgical margins on fear of cancer recurrence: results from CaPSURE Urol Oncol 28: 268-273

65. Ilie G, White J, Mason R, Rendon R, Bailly G, Lawen J, Bowes D, Patil N, Wilke D, MacDonald C, Rutledge R, Bell D (2020) Current Mental Distress Among Men With a History of Radical Prostatectomy and Related Adverse Correlates Am J Mens Health 14: 1557988320957535

66. Isaka S (1996) [QOL assessment in the treatment of prostate cancer] Gan To Kagaku Ryoho 23: 427-431

67. Jenkins R, Schover LR, Fouladi RT, Warneke C, Neese L, Klein EA, Zippe C, Kupelian P (2004) Sexuality and health-related quality of life after prostate cancer in African-American and white men treated for localized disease J Sex Marital Ther 30: 79-93

68. Jones SM, Ziebell R, Walker R, Nekhlyudov L, Rabin BA, Nutt S, Fujii M, Chubak J (2017) Association of worry about cancer to benefit finding and functioning in long-term cancer survivors Support Care Cancer 25: 1417-1422

69. Joshy G, Thandrayen J, Koczwara B, Butow P, Laidsaar-Powell R, Rankin N, Canfell K, Stubbs J, Grogan P, Bailey L, Yazidjoglou A, Banks E (2020) Disability, psychological distress and quality of life in relation to cancer diagnosis and cancer type: population-based Australian study of 22,505 cancer survivors and 244,000 people without cancer BMC Med 18: 372

70. Kim SH, Seong DH, Yoon SM, Choi YD, Choi E, Song H (2017) Predictors of health-related quality of life in Korean prostate cancer patients receiving androgen deprivation therapy Eur J Oncol Nurs 30: 84-90

71. Kim Y, Kashy DA, Wellisch DK, Spillers RL, Kaw CK, Smith TG (2008) Quality of life of couples dealing with cancer: dyadic and individual adjustment among breast and prostate cancer survivors and their spousal caregivers Ann Behav Med 35: 230-238

72. Kirschner-Hermanns R, Jakse G (2002) Quality of life following radical prostatectomy Crit Rev Oncol Hematol 43: 141-151

73. Klotz L (2013) Active Surveillance, Quality of Life, and Cancer-related Anxiety European Urology 64: 37-39

74. Knapp K, Cooper B, Koetters T, Cataldo J, Dhruva A, Paul SM, West C, Aouizerat BE, Miaskowski C (2012) Trajectories and predictors of symptom occurrence, severity, and distress in prostate cancer patients undergoing radiation therapy J Pain Symptom Manage 44: 486-507

75. Koch L, Jansen L, Brenner H, Arndt V (2013) Fear of recurrence and disease progression in long-term (≥5 years) cancer survivors—a systematic review of quantitative studies Psycho-Oncology 22: 1-11

76. Kornblith AB, Herr HW, Ofman US, Scher HI, Holland JC (1994) Quality of life of patients with prostate cancer and their spouses. The value of a data base in clinical care Cancer 73: 2791-2802

77. Kypriotakis G, Deimling GT, Piccinin AM, Hofer SM (2016) Correlated and Coupled Trajectories of Cancer-Related Worries and Depressive Symptoms among Long-Term Cancer Survivors Behavioral Medicine 42: 82-92

78. Latini DM, Hart SL, Knight SJ, Cowan JE, Ross PL, Duchane J, Carroll PR (2007) The relationship between anxiety and time to treatment for patients with prostate cancer on surveillance J Urol 178: 826-831; discussion 831-822

79. Lebel S, Simard S, Harris C, Feldstain A, Beattie S, McCallum M, Lefebvre M, Savard J, Devins GM (2016) Empirical validation of the English version of the Fear of Cancer Recurrence Inventory Quality of Life Research 25: 311-321

80. Lebel S, Tomei C, Feldstain A, Beattie S, McCallum M (2013) Does fear of cancer recurrence predict cancer survivors' health care use? Supportive Care in Cancer 21: 901-906

81. Lilleby W, Fosså SD, Waehre HR, Olsen DR (1999) Long-term morbidity and quality of life in patients with localized prostate cancer undergoing definitive radiotherapy or radical prostatectomy Int J Radiat Oncol Biol Phys 43: 735-743

82. Loeb S, Curnyn C, Walter D, Fagerlin A, Siebert U, Mühlberger N, Braithwaite RS, Schwartz MD, Lepor H, Sedlander E (2018) Health state utilities among contemporary prostate cancer patients on active surveillance Transl Androl Urol 7: 197-202

83. Lubeck DP, Kim H, Grossfeld G, Ray P, Penson DF, Flanders SC, Carroll PR (2001) Health related quality of life differences between black and white men with prostate cancer: data from the cancer of the prostate strategic urologic research endeavor J Urol 166: 2281-2285

84. Maggi M, Gentilucci A, Salciccia S, Gatto A, Gentile V, Colarieti A, Von Heland M, Busetto GM, Del Giudice F, Sciarra A (2019) Psychological impact of different primary treatments for prostate cancer: A critical analysis Andrologia 51: e13157

85. Maguire R, Hanly P, Drummond FJ, Gavin A, Sharp L (2017) Regret and fear in prostate cancer: The relationship between treatment appraisals and fear of recurrence in prostate cancer survivors Psychooncology 26: 1825-1831

86. McGinty H, Flury S, Yanez B, Buckman V, Penedo F (2015) Coping style mediates the relationship between symptom burden and cancer specific distress in men with advanced prostate cancer.

87. Mehnert A, Koch U (2008) Psychometric evaluation of the German version of the Life Attitude Profile-Revised (LAP-R) in prostate cancer patients Palliat Support Care 6: 119-124

88. Mehnert A, Lehmann C, Graefen M, Huland H, Koch U (2010) Depression, anxiety, post-traumatic stress disorder and health-related quality of life and its association with social support in ambulatory prostate cancer patients Eur J Cancer Care (Engl) 19: 736-745

89. Mellon S, Kershaw TS, Northouse LL, Freeman-Gibb L (2007) A family-based model to predict fear of recurrence for cancer survivors and their caregivers Psychooncology 16: 214-223

90. Meropol NJ, Egleston BL, Buzaglo JS, Benson Iii AB, Cegala DJ, Diefenbach MA, Fleisher L, Miller SM, Sulmasy Ofm DP, Weinfurt KP, Group CSR (2008) Cancer patient preferences for quality and length of life Cancer 113: 3459-3466

91. Müller G, Otto U (2015) [Quality of life, psychological distress, and social outcomes after radical prostatectomy. Results from a urology competence center for rehabilitation] Urologe A 54: 1555-1563

92. Nelson CJ, Starr TD, Macchia RJ, Hyacinthe L, Friedman S, Roth AJ (2016) Assessing anxiety in Black men with prostate cancer: further data on the reliability and validity of the Memorial Anxiety Scale for Prostate Cancer (MAX-PC) Support Care Cancer 24: 2905-2911

93. Nelson CJ, Weinberger MI, Balk E, Holland J, Breitbart W, Roth AJ (2009) The chronology of distress, anxiety, and depression in older prostate cancer patients Oncologist 14: 891-899

94. Oba A, Nakaya N, Saito-Nakaya K, Hasumi M, Takechi H, Arai S, Shimizu N (2017) Psychological distress in men with prostate cancer and their partners before and after cancer diagnosis: a longitudinal study Jpn J Clin Oncol 47: 735-742

95. Occhipinti S, Zajdlewicz L, Coughlin GD, Yaxley JW, Dunglison N, Gardiner RA, Chambers SK (2019) A prospective study of psychological distress after prostate cancer surgery Psychooncology 28: 2389-2395

96. Ottenbacher A, Sloane R, Snyder DC, Kraus W, Sprod L, Demark-Wahnefried W (2013) Cancer-specific concerns and physical activity among recently diagnosed breast and prostate cancer survivors Integr Cancer Ther 12: 206-212

97. P. D, L. DL, S. D, B. A, M. C, T. M, C. M, B.N. C (2020) The relationship between prostate cancer anxiety and mindfulness ability in patients with PCA, Abstracts of the 30th annual meeting of the italian society of uro-oncology (siuro) Anticancer Research 40: 4571

98. Parker A, Tavlarides A, McNeil R, Green K, Ames S, Igel T (2010) 1958 pretreatment psa level and gleason score are not associated with cancer-specific anxiety prior to surgical treatment for newly diagnosed prostate cancer.

99. Pastore AL, Mir A, Maruccia S, Palleschi G, Carbone A, Lopez C, Camps N, Palou J (2017) Psychological distress in patients undergoing surgery for urological cancer: A single centre cross-sectional study Urol Oncol 35: 673.e671-673.e677

100. Perczek RE, Burke MA, Carver CS, Krongrad A, Terris MK (2002) Facing a prostate cancer diagnosis: who is at risk for increased distress? Cancer 94: 2923-2929

101. Philip EJ, Bergerot CD, Clark K, Bergerot P, Loscalzo M (2019) Obesity and psychosocial well-being among cancer patients and survivors Psychooncology 28: 2141-2148

102. Potosky AL, Knopf K, Clegg LX, Albertsen PC, Stanford JL, Hamilton AS, Gilliland FD, Eley JW, Stephenson RA, Hoffman RM (2001) Quality-of-life outcomes after primary androgen deprivation therapy: results from the Prostate Cancer Outcomes Study J Clin Oncol 19: 3750-3757

103. Ream E, Quennell A, Fincham L, Faithfull S, Khoo V, Wilson-Barnett J, Richardson A (2008) Supportive care needs of men living with prostate cancer in England: a survey Br J Cancer 98: 1903-1909

104. Resnick MJ, Guzzo TJ, Cowan JE, Knight SJ, Carroll PR, Penson DF (2013) Factors associated with satisfaction with prostate cancer care: results from Cancer of the Prostate Strategic Urologic Research Endeavor (CaPSURE) BJU International 111: 213-220

105. Roeloffzen EMA, Lips IM, van Gellekom MPR, van Roermund J, Frank SJ, Battermann JJ, van Vulpen M (2010) Health-Related Quality of Life up to Six Years After 125I Brachytherapy for Early-Stage Prostate Cancer International Journal of Radiation Oncology*Biology*Physics 76: 1054-1060

106. Rönningås U, Fransson P, Holm M, Wennman-Larsen A (2019) Prostate-specific antigen (PSA) and distress: - a cross-sectional nationwide survey in men with prostate cancer in Sweden BMC Urol 19: 66

107. Schouten B, Avau B, Bekkering GTE, Vankrunkelsven P, Mebis J, Hellings J, Van Hecke A (2019) Systematic screening and assessment of psychosocial well-being and care needs of people with cancer Cochrane Database Syst Rev 3: Cd012387

108. Séguin Leclair C, Lebel S, Westmaas JL (2019) The relationship between fear of cancer recurrence and health behaviors: A nationwide longitudinal study of cancer survivors Health Psychol 38: 596-605

109. Seiler D, Randazzo M, Leupold U, Zeh N, Isbarn H, Chun FK, Ahyai SA, Baumgartner M, Huber A, Recker F, Kwiatkowski M (2012) Protocol-based active surveillance for low-risk prostate cancer: anxiety levels in both men and their partners Urology 80: 564-569

110. Sharp L, O'Leary E, Kinnear H, Gavin A, Drummond FJ (2016) Cancer-related symptoms predict psychological wellbeing among prostate cancer survivors: results from the PiCTure study Psychooncology 25: 282-291

111. Smith D, Egger S, O'Connell D, King M, Stricker P, Cozzi P, Berry M, Armstrong B (2015) Ten-year quality of life and psychological outcomes of men managed entirely with active surveillance: The NSW Prostate Cancer Care and Outcomes Study.

112. Smith TG, Strollo S, Hu X, Earle CC, Leach CR, Nekhlyudov L (2019) Understanding Long-Term Cancer Survivors’ Preferences for Ongoing Medical Care Journal of General Internal Medicine 34: 2091-2097

113. Soloway CT, Soloway MS, Kim SS, Kava BR (2005) Sexual, psychological and dyadic qualities of the prostate cancer 'couple' BJU Int 95: 780-785

114. Tan HJ, Marks LS, Hoyt MA, Kwan L, Filson CP, Macairan M, Lieu P, Litwin MS, Stanton AL (2016) The Relationship between Intolerance of Uncertainty and Anxiety in Men on Active Surveillance for Prostate Cancer J Urol 195: 1724-1730

115. Torbit LA, Albiani JJ, Crangle CJ, Latini DM, Hart TL (2015) Fear of recurrence: the importance of self-efficacy and satisfaction with care in gay men with prostate cancer Psycho-Oncology 24: 691-698

116. Traeger L, Penedo FJ, Gonzalez JS, Dahn JR, Lechner SC, Schneiderman N, Antoni MH (2009) Illness perceptions and emotional well-being in men treated for localized prostate cancer J Psychosom Res 67: 389-397

117. Turner EL, Lane JA, Metcalfe C, Down L, Donovan JL, Hamdy F, Neal D, Vedhara K (2009) Psychological distress and prostate specific antigen levels in men with and without prostate cancer Brain Behav Immun 23: 1073-1078

118. Ullrich PM, Carson MR, Lutgendorf SK, Williams RD (2003) Cancer fear and mood disturbance after radical prostatectomy: consequences of biochemical evidence of recurrence J Urol 169: 1449-1452

119. van den Bergh RC, Korfage IJ, Borsboom GJ, Steyerberg EW, Essink-Bot ML (2009) Prostate cancer-specific anxiety in Dutch patients on active surveillance: validation of the memorial anxiety scale for prostate cancer Qual Life Res 18: 1061-1066

120. van Stam MA, Aaronson NK, Bosch J, Kieffer JM, van der Voort van Zyp JRN, Tillier CN, Horenblas S, van der Poel HG (2020) Patient-reported Outcomes Following Treatment of Localised Prostate Cancer and Their Association with Regret About Treatment Choices Eur Urol Oncol 3: 21-31

121. Vasarainen H, Lokman U, Ruutu M, Taari K, Rannikko A (2012) Prostate cancer active surveillance and health-related quality of life: results of the Finnish arm of the prospective trial BJU Int 109: 1614-1619

122. Venderbos L, van den Bergh R, Bangma C, Roobol M (2019) Eight year patient reported outcome data of the first 150 Dutch men on active surveillance in the Prostate cancer Research International Active Surveillance study (PRIAS) European Urology Supplements 18: e1707-e1708

123. Victorson David E, Schuette S, Schalet Benjamin D, Kundu Shilajit D, Helfand Brian T, Novakovic K, Sufrin N, McGuire M, Brendler C (2016) Factors Affecting Quality of Life at Different Intervals After Treatment of Localized Prostate Cancer: Unique Influence of Treatment Decision Making Satisfaction, Personality and Sexual Functioning Journal of Urology 196: 1422-1428

124. Visser A, van Andel G, Willems P, Voogt E, Dijkstra A, Rovers P, Goodkin K, Kurth KH (2003) Changes in health-related quality of life of men with prostate cancer 3 months after diagnosis: the role of psychosocial factors and comparisment with benign prostate hyperplasia patients Patient Educ Couns 49: 225-232

125. Wallerstedt A, Nyberg T, Carlsson S, Thorsteinsdottir T, Stranne J, Tyritzis SI, Stinesen Kollberg K, Hugosson J, Bjartell A, Wilderäng U, Wiklund P, Steineck G, Haglind E (2019) Quality of Life After Open Radical Prostatectomy Compared with Robot-assisted Radical Prostatectomy Eur Urol Focus 5: 389-398

126. Watson E, Shinkins B, Frith E, Neal D, Hamdy F, Walter F, Weller D, Wilkinson C, Faithfull S, Wolstenholme J, Sooriakumaran P, Kastner C, Campbell C, Neal R, Butcher H, Matthews M, Perera R, Rose P (2016) Symptoms, unmet needs, psychological well-being and health status in survivors of prostate cancer: implications for redesigning follow-up BJU Int 117: E10-19

127. White K, D'Abrew N, Katris P, O'Connor M, Emery L (2012) Mapping the psychosocial and practical support needs of cancer patients in Western Australia European Journal of Cancer Care 21: 107-116

128. Wilcox CB, Gilbourd D, Louie-Johnsun M (2014) Anxiety and health-related quality of life (HRQL) in patients undergoing active surveillance of prostate cancer in an Australian centre BJU International 113: 64-68

129. Wilding S, Downing A, Wright P, Selby P, Watson E, Wagland R, Donnelly DW, Hounsome L, Butcher H, Mason M, Henry A, Gavin A, Glaser AW (2019) Cancer-related symptoms, mental well-being, and psychological distress in men diagnosed with prostate cancer treated with androgen deprivation therapy Qual Life Res 28: 2741-2751

130. Wollersheim BM, van Asselt KM, van der Poel HG, van Weert H, Hauptmann M, Retèl VP, Aaronson NK, van de Poll-Franse LV, Boekhout AH (2020) Design of the PROstate cancer follow-up care in Secondary and Primary hEalth Care study (PROSPEC): a randomized controlled trial to evaluate the effectiveness of primary care-based follow-up of localized prostate cancer survivors BMC Cancer 20: 635

131. Wu LM, McGinty H, Amidi A, Bovbjerg K, Diefenbach MA (2019) Longitudinal dyadic associations of fear of cancer recurrence and the impact of treatment in prostate cancer patients and their spouses Acta Oncologica 58: 708-714

132. Yanez B, Bustillo NE, Antoni MH, Lechner SC, Dahn J, Kava B, Penedo FJ (2015) The importance of perceived stress management skills for patients with prostate cancer in active surveillance J Behav Med 38: 214-223

133. Zajdlewicz L, Hyde MK, Lepore SJ, Gardiner RA, Chambers SK (2017) Health-Related Quality of Life After the Diagnosis of Locally Advanced or Advanced Prostate Cancer: A Longitudinal Study Cancer Nurs 40: 412-419
