# Supplementary material for: Fear of cancer recurrence and PSA anxiety in patients with prostate cancer: a systematic review
Source: Support Care Cancer. 2022 Feb 1;30(7):5577–89. doi: 10.1007/s00520-022-06876-z (PMC9135793; doi:10.1007/s00520-022-06876-z)
Supplement: Supplementary file 3 — Supplementary file3 (DOCX 57 KB) [file 520_2022_6876_MOESM3_ESM.docx]

**Online resource 3: full study characteristics**

| **Author** | **Year** | **Country** | **Sample size** | **Sample mean age** | **Pre/post treatment** | **Study type** | **Time point** | **Treatment** | **Tool used** | **Event cutoff** | |
| --- | --- | --- | --- | --- | --- | --- | --- | --- | --- | --- | --- |
| Alvisi et al(1) | 2020 | Italy | 236 | 64 | N/A | Longitudinal | 0-10 months | AS | MAXPC | FCR:≥4.5  PSA anxiety: ≥3 | |
| Anderson et al(2) | 2014 | Australia | 86 | 65.7 | N/A | Cross sectional | 22 months (mean) | AS | MAXPC | >7 FOR, >5 PSA | |
| Belizzi et al (3) | 2007 | USA | 730 | 63.2 | Pre and post | Longitudinal | 9 months before treatment to 1 year after | RP, RT | Kornblith’s 5-item Fear of Recurrence Scale | N/A | |
| Chambers et al (4) | 2017 | Australia | 1064 | 63.7 | Post | Longitudinal | 6 years (mean) | Mixed | MaxPc | N/A | |
| Chien et al (5) | 2018 | Taiwan | 48 | 67 | Post | Longitudinal | 0-6 months (mean) | RP, RT | MAXPC | N/A | |
| clark et al (6) | 2006 | USA | 235 | Median: 64 | Post | Longitudinal | 6 years (mean) | Mixed | Previous study | N/A | |
| Meissner et al(7) | 2017 | Germany | 4719 | 75.2 | Post | Longitudinal | 11.5 years mean | RP | MAXPC | >6 | |
| Dowrick et al (8) | 2018 | Australia | 540 | 62 | Post | Longitudinal | 1 year (mean) | RP | Clark cancer wory psa concer subscale | N/A | |
| Egger et al (9) | 2018 | Australia | 278 | 69 | Post | Longitudinal | 9.8 years mean | Mixed | Kornblith FOP | N/A | |
| Eisenberg et al (10) | 2014 | USA | 67 | 64.3 | Post | Longitudinal | 36-60 months | Mixed | Fear of Recurrence Scale | Not listed | |
| Ettridge et al (11) | 2020 | Australia | 276 | 66.3 | Pre and post | Longitudinal | Pre treatment, 6,12 and 24 months | Mixed | MAXPC | N/A | |
| Götze et al (12) | 2019 | Germany | 255 | Not given | Post | Longitudinal | 7.5 years (mean) | Mixed | FOP-Q | >34 | |
| Hart et al (13) | 2014 | Canada | 92 | 57.8 (Gay men) | Post | Cross sectional | 1.9 years (mean) | Mixed | Kornblith FOR | N/A | |
| Huang et al (14) | 2017 | China | 254 | 68.25 | Pre and post | Cross sectional | Not stated | Mixed | MAX PC | N/A | |
| Kendel et al (15) | 2016 | Germany | 370 | 66.6 | Post | Cross sectional | 4 years (mean) | Mixed | MAXPC FOR | N/A | |
| Koch-Gallenkamp et al (16) | 2016 | Germany | 2092 | 69 | post | Cross sectional | Not stated | Mixed | FOPQ | >4 or equal to, in 50 % of questions | |
| Mehta et al (17) | 2003 | United States | 519 | RP=61.2, RT= 69.3, BT=71.6 | Pre and post | Longitudinal | 0-2 years | RP,RT,BT | FCR scale | N/A | |
| Mehnert et al (18) | 2007 | Germany | 197 | 65.9 | Post | Cross sectional | 10 months (mean) | RP | MAXPC |  | |
| Mahal et al (19) | 2015 | USA | 375 | N/A | Post | Cross sectional | 5.1 years (mean) | RT, BT | MAXPC PSA | ≥2 in any question | |
| Meissner et al(20) | 2021 | Germany | 2417 | 69.5 | Post | Longitudinal | 7 years, 16 years | RP | Fop-q | ≥34 | |
| Naha et al (21) | 2020 | USA | 2162 | 65 | Post | Longitudinal | 0-36 months | AS | MAXPC | N/A | |
| Nillson et al (22) | 2021 | Norway | 440 | 65.9 | Post | Cross sectional | 4.1 years (mean) | AS, RP | CARQ | ≥12 | |
| Roth et al (23) | 2003 | USA | 385 | 71.1 | Post | Cross sectional | N/A | Mixed | MAXPC | N/A | |
| Savard et al (24) | 2018 | Canada | 263 | Not available | Pre and post | Longitudinal | [0-18](https://www.sciencedirect.com/topics/medicine-and-dentistry/perioperative-period) months | Any | FCRI | ≥13 | |
| Sevier Guy et al(25) | 2021 | UK | 144 | 68 | Post | Cross sectional | 6 years | Mixed | FCR7 | N/A | |
| Tavlarides et al (26) | 2012 | USA | 365 | 63.9 | Post | Cross sectional | 12 months | RP | MAXPC | N/A | |
| Touzani et al (27) | 2019 | France | 185 | 65.1 | Post | Cross sectional | Not stated | Any | MAXPC | N/A | |
| Ussher et al (28) | 2016 | Australia +UK/US | GB=119,H=224 | GB 64.2, H (hererosexual)=71.5 | Post | Cross sectional | GB=5.9 years, H=7.7 years (means) | Any | MAXPC | N/A | |
| Parker et al (29) | 2016 | USA | 180 | 67.2 | Post | Longitudinal | 0-30 months | AS | MAXPC-FOR | N/A | |
| van den Bergh et al (30) | 2010 | Netherlands | 129 | 64.6 | Post | Longitudinal | 6-18 months | AS | MAXPC+ self-estimated disease progression | N/A | |
| van de Wal et al (31) | 2016 | Netherlands | 283 | 62.5 at diagnosis, 70 at survey | Post | Cross sectional | 7.1 years (mean) | RT,RP | Cancer Worry Scale: FCR severity, MAXPC PSA | ≥to 14 CWS | |
| Villa ET AL (32) | 2015 | Italy | 207 | Not available | POST/PRE | Longitudinal | 0-19 months | AS | MAXPC | N/A | |
| Index: ADT= androgen deprivation therapy AS= active surveillance, BT= brachytherapy, FCR= fear of cancer recurrence, FOP= fear of progression, FoR= fear of recurrence, GB= gay or bisexual, H= Heterosexual, HT= hormone therapy MAXPC= memorial anxiety scale for prostate cancer, RP= radical prostatectomy, RT= radiation therapy | | | | | | | | | | |  |

**References**

1. Alvisi MF, Dordoni P, Rancati T, Avuzzi B, Nicolai N, Badenchini F, et al. Supporting Patients With Untreated Prostate Cancer on Active Surveillance: What Causes an Increase in Anxiety During the First 10 Months? Front Psychol. 2020;11:576459.

2. Anderson J, Burney S, Brooker JE, Ricciardelli LA, Fletcher JM, Satasivam P, et al. Anxiety in the management of localised prostate cancer by active surveillance. BJU International. 2014;114(S1):55-61.

3. Bellizzi KM, Latini DM, Cowan JE, DuChane J, Carroll PR. Fear of recurrence, symptom burden, and health-related quality of life in men with prostate cancer. Urology. 2008;72(6):1269-73.

4. Chambers SK, Ng SK, Baade P, Aitken JF, Hyde MK, Wittert G, et al. Trajectories of quality of life, life satisfaction, and psychological adjustment after prostate cancer. Psychooncology. 2017;26(10):1576-85.

5. Chien CH, Chuang CK, Liu KL, Wu CT, Pang ST, Chang YH. Positive and negative affect and prostate cancer-specific anxiety in Taiwanese patients and their partners. Eur J Oncol Nurs. 2018;37:1-11.

6. Clark JA, Talcott JA. Confidence and uncertainty long after initial treatment for early prostate cancer: survivors' views of cancer control and the treatment decisions they made. J Clin Oncol. 2006;24(27):4457-63.

7. Meissner VH, Herkommer K, Marten-Mittag B, Gschwend JE, Dinkel A. Prostate cancer-related anxiety in long-term survivors after radical prostatectomy. J Cancer Surviv. 2017;11(6):800-7.

8. Dowrick AS, Wootten AC, Botti M. Does partnership status affect the quality of life of men having robotic-assisted radical prostatectomy (RARP) for localised prostate cancer? Applied Nursing Research. 2018;42:51-5.

9. Egger SJ, Calopedos RJ, O'Connell DL, Chambers SK, Woo HH, Smith DP. Long-term Psychological and Quality-of-life Effects of Active Surveillance and Watchful Waiting After Diagnosis of Low-risk Localised Prostate Cancer. Eur Urol. 2018;73(6):859-67.

10. Eisenberg SA, Kurita K, Taylor-Ford M, Agus DB, Gross ME, Meyerowitz BE. Intolerance of uncertainty, cognitive complaints, and cancer-related distress in prostate cancer survivors. Psychooncology. 2015;24(2):228-35.

11. Ettridge K, Wright K, Smith D, Chambers S, Corsini N, Evans S, et al. Measuring psychosocial outcomes of men living with prostate cancer: feasibility of regular assessment of patient-reported outcomes. Eur J Cancer Care (Engl). 2020:e13393.

12. Götze H, Taubenheim S, Dietz A, Lordick F, Mehnert-Theuerkauf A. Fear of cancer recurrence across the survivorship trajectory: Results from a survey of adult long-term cancer survivors. Psychooncology. 2019;28(10):2033-41.

13. Hart TL, Coon DW, Kowalkowski MA, Zhang K, Hersom JI, Goltz HH, et al. Changes in sexual roles and quality of life for gay men after prostate cancer: challenges for sexual health providers. J Sex Med. 2014;11(9):2308-17.

14. Huang Q, Jiang P, Zhang Z, Luo J, Dai Y, Zheng L, et al. Assessing cancer-specific anxiety in Chinese men with prostate cancer: psychometric evaluation of the Chinese version of the Memorial Anxiety Scale for Prostate Cancer (MAX-PC). Support Care Cancer. 2017;25(12):3683-90.

15. Kendel F, Helbig L, Neumann K, Herden J, Stephan C, Schrader M, et al. Patients' perceptions of mortality risk for localized prostate cancer vary markedly depending on their treatment strategy. Int J Cancer. 2016;139(4):749-53.

16. Koch-Gallenkamp L, Bertram H, Eberle A, Holleczek B, Schmid-Höpfner S, Waldmann A, et al. Fear of recurrence in long-term cancer survivors-Do cancer type, sex, time since diagnosis, and social support matter? Health Psychol. 2016;35(12):1329-33.

17. Mehta SS, Lubeck DP, Pasta DJ, Litwin MS. Fear of Cancer Recurrence in Patients Undergoing Definitive Treatment for Prostate Cancer: Results From CaPSURE. The Journal of Urology. 2003;170(5):1931-3.

18. Mehnert A, Lehmann C, Schulte T, Koch U. Presence of symptom distress and prostate cancer-related anxiety in patients at the beginning of cancer rehabilitation. Onkologie. 2007;30(11):551-6.

19. Mahal BA, Chen MH, Bennett CL, Kattan MW, Sartor O, Stein K, et al. High PSA anxiety and low health literacy skills: drivers of early use of salvage ADT among men with biochemically recurrent prostate cancer after radiotherapy? Ann Oncol. 2015;26(7):1390-5.

20. Meissner VH, Olze L, Schiele S, Ankerst DP, Jahnen M, Gschwend JE, et al. Fear of cancer recurrence and disease progression in long-term prostate cancer survivors after radical prostatectomy: A longitudinal study. Cancer.n/a(n/a).

21. Naha U, Freedland SJ, Abern MR, Moreira DM. The association of cancer-specific anxiety with disease aggressiveness in men on active surveillance of prostate cancer. Prostate Cancer Prostatic Dis. 2020.

22. Nilsson R, Næss-Andresen TF, Myklebust TÅ, Bernklev T, Kersten H, Haug ES. Fear of Recurrence in Prostate Cancer Patients: A Cross-sectional Study After Radical Prostatectomy or Active Surveillance. European Urology Open Science. 2021;25:44-51.

23. Roth AJ, Rosenfeld B, Kornblith AB, Gibson C, Scher HI, Curley-Smart T, et al. The Memorial Anxiety Scale for Prostate Cancer. Cancer. 2003;97(11):2910-8.

24. Savard J, Ivers H. The evolution of fear of cancer recurrence during the cancer care trajectory and its relationship with cancer characteristics. Journal of Psychosomatic Research. 2013;74(4):354-60.

25. Sevier-Guy L-J, Ferreira N, Somerville C, Gillanders D. Psychological flexibility and fear of recurrence in prostate cancer. European Journal of Cancer Care.n/a(n/a):e13483.

26. Tavlarides AM, Ames SC, Diehl NN, Joseph RW, Castle EP, Thiel DD, et al. Evaluation of the association of prostate cancer-specific anxiety with sexual function, depression and cancer aggressiveness in men 1 year following surgical treatment for localized prostate cancer. Psychooncology. 2013;22(6):1328-35.

27. Touzani R, Mancini J, Troïan J, Bouhnik AD, Cussenot O, Gravis G, et al. Adaptation and validation of the memorial anxiety scale for prostate cancer (MAX-PC) in a sample of French men. J Patient Rep Outcomes. 2019;3(1):60.

28. Ussher JM, Perz J, Kellett A, Chambers S, Latini D, Davis ID, et al. Health-Related Quality of Life, Psychological Distress, and Sexual Changes Following Prostate Cancer: A Comparison of Gay and Bisexual Men with Heterosexual Men. J Sex Med. 2016;13(3):425-34.

29. Parker PA, Davis JW, Latini DM, Baum G, Wang X, Ward JF, et al. Relationship between illness uncertainty, anxiety, fear of progression and quality of life in men with favourable-risk prostate cancer undergoing active surveillance. BJU Int. 2016;117(3):469-77.

30. van den Bergh RC, Essink-Bot ML, Roobol MJ, Schröder FH, Bangma CH, Steyerberg EW. Do anxiety and distress increase during active surveillance for low risk prostate cancer? J Urol. 2010;183(5):1786-91.

31. van de Wal M, van Oort I, Schouten J, Thewes B, Gielissen M, Prins J. Fear of cancer recurrence in prostate cancer survivors. Acta Oncol. 2016;55(7):821-7.

32. Villa S, Repetto C, Rancati T, Avuzzi B, Catanzaro M, Marenghi C, et al. Prostate cancer-related anxiety: From enrolment to one year after the first re-biopsy. Anticancer Research. 2015;35(6):3671-2.
