# Supplementary material for: Fear of cancer recurrence and PSA anxiety in patients with prostate cancer: a systematic review
Source: Support Care Cancer. 2022 Feb 1;30(7):5577–89. doi: 10.1007/s00520-022-06876-z (PMC9135793; doi:10.1007/s00520-022-06876-z)
Supplement: Supplementary file 4 — Supplementary file4 (DOCX 16 KB) [file 520_2022_6876_MOESM4_ESM.docx]

**Online resource 4 JBI risk of bias for cross sectional studies adapted from JBI risk of bias (1)**

| Risk of bias item | Score |
| --- | --- |
| Were the criteria for inclusion in the sample clearly defined? | Yes=1  No=0  Not applicable=N/A |
| Were the study subjects and the setting described in detail? | Yes=1  No=0  Not applicable=N/A |
| Was the exposure measured in a valid and reliable way? | Yes=1  No=0  Not applicable=N/A |
| Were objective, standard criteria used for measurement of the condition? | Yes=1  No=0  Not applicable=N/A |
| Were confounding factors identified? | Yes=1  No=0  Not applicable=N/A |
| Were strategies to deal with confounding factors stated? | Yes=1  No=0  Not applicable=N/A |
| Were the outcomes measured in a valid and reliable way? | Yes=1  No=0  Not applicable=N/A |
| Was appropriate statistical analysis used? | Yes=1  No=0  Not applicable=N/A |

**Supplementary Table A4 JBI risk of bias for longitudinal studies adapted from (1)**

| Risk of bias item | Score |
| --- | --- |
| Were the two groups similar and recruited from the same population? | Yes=1  No=0  Not applicable=N/A |
| Were the exposures measured similarly to assign people to both exposed and unexposed groups? | Yes=1  No=0  Not applicable=N/A |
| Was the exposure measured in a valid and reliable way? | Yes=1  No=0  Not applicable=N/A |
| Were confounding factors identified? | Yes=1  No=0  Not applicable=N/A |
| Were strategies to deal with confounding factors stated? | Yes=1  No=0  Not applicable=N/A |
| Were the groups/participants free of the outcome at the start of the study (or at the moment of exposure)? | Yes=1  No=0  Not applicable=N/A |
| Were the outcomes measured in a valid and reliable way? | Yes=1  No=0  Not applicable=N/A |
| Was the follow up time reported and sufficient to be long enough for outcomes to occur? | Yes=1  No=0  Not applicable=N/A |
| Was follow up complete, and if not, were the reasons to loss to follow up described and explored? | Yes=1  No=0  Not applicable=N/A |
| Were strategies to address incomplete follow up utilized? | Yes=1  No=0  Not applicable=N/A |
| Was appropriate statistical analysis used? | Yes=1  No=0  Not applicable=N/A |

**References**

1. JBI. JBI Manual for evidence synthesis. 2020.
