# Supplementary material for: Fear of cancer recurrence and PSA anxiety in patients with prostate cancer: a systematic review
Source: Support Care Cancer. 2022 Feb 1;30(7):5577–89. doi: 10.1007/s00520-022-06876-z (PMC9135793; doi:10.1007/s00520-022-06876-z)
Supplement: Supplementary file 5 — Supplementary file5 (DOCX 38 KB) [file 520_2022_6876_MOESM5_ESM.docx]

**Online resource 5: Risk of bias results for cross sectional studies**

| Author | Inclusion criteria | study subjects and the setting | exposure | Objective, standard criteria used for measurement of the condition | confounding factors | strategies to deal with confounding factors | outcomes measured in a valid and reliable way | appropriate statistical analysis used | Score out of 8 | Risk of bias |
| --- | --- | --- | --- | --- | --- | --- | --- | --- | --- | --- |
| Anderson et al(1) | 1 | 1 | 1 | 1 | 0 | 0 | 1 | 1 | 6 | Moderate |
| (2)Hart et al | 1 | 0 | 1 | 1 | 0 | 0 | 1 | 1 | 5 | Moderate |
| Huang et al (3) | 1 | 1 | 1 | 1 | 1 | 1 | 1 | 1 | 8 | Low |
| Jones et al (4) | 1 | 1 | 0 | 0 | 0 | 0 | 1 | 1 | 4 | High |
| Kendel et al (5) | 1 | 1 | 1 | 1 | 0 | 0 | 1 | 1 | 6 | Moderate |
| Koch Gallenkamp et al (6) | 1 | 1 | 0 | 1 | 0 | 0 | 1 | 1 | 5 | Moderate |
| Lebel et al (7) | 1 | 1 | 0 | 0 | 0 | 0 | 1 | 1 | 4 | High |
| Maguire et al (8) | 1 | 1 | 0 | 0 | 0 | 0 | 1 | 1 | 4 | High |
| Mahal et al (9) | 1 | 1 | 1 | 1 | 0 | 0 | 1 | 1 | 6 | Moderate |
| Mehnert et al (10) | 1 | 1 | 1 | 1 | 1 | 0 | 1 | 1 | 5 | Moderate |
| Mehta et al(11) | 1 | 1 | 1 | 0 | 0 | 1 | 1 | 1 | 6 | Moderate |
| Nillson et al (12) | 1 | 1 | 1 | 1 | 0 | 0 | 1 | 1 | 6 | Moderate |
| Roth et al (13) | 1 | 1 | 1 | 1 | 0 | 0 | 1 | 1 | 6 | Moderate |
| Sevier Guy et al (14) | 1 | 1 | 1 | 1 | 0 | 1 | 1 | 1 | 7 | Low |
| Tavlarides et al (15) | 1 | 1 | 1 | 1 | 1 | 1 | 1 | 1 | 8 | Low |
| Touzani et al (16) | 1 | 1 | 1 | 0 | 0 | 0 | 1 | 1 | 5 | Moderate |
| Ussher e al (17) | 1 | 0 | 1 | 1 | 0 | 0 | 1 | 1 | 5 | Moderate |
| van de Wal et al (18) | 1 | 1 | 1 | 1 | 0 | 0 | 1 | 1 | 6 | Moderate |

**References**

1. Anderson J, Burney S, Brooker JE, Ricciardelli LA, Fletcher JM, Satasivam P, et al. Anxiety in the management of localised prostate cancer by active surveillance. BJU International. 2014;114(S1):55-61.

2. Hart TL, Coon DW, Kowalkowski MA, Zhang K, Hersom JI, Goltz HH, et al. Changes in sexual roles and quality of life for gay men after prostate cancer: challenges for sexual health providers. J Sex Med. 2014;11(9):2308-17.

3. Huang Q, Jiang P, Zhang Z, Luo J, Dai Y, Zheng L, et al. Assessing cancer-specific anxiety in Chinese men with prostate cancer: psychometric evaluation of the Chinese version of the Memorial Anxiety Scale for Prostate Cancer (MAX-PC). Support Care Cancer. 2017;25(12):3683-90.

4. Jones SM, Ziebell R, Walker R, Nekhlyudov L, Rabin BA, Nutt S, et al. Association of worry about cancer to benefit finding and functioning in long-term cancer survivors. Support Care Cancer. 2017;25(5):1417-22.

5. Kendel F, Helbig L, Neumann K, Herden J, Stephan C, Schrader M, et al. Patients' perceptions of mortality risk for localized prostate cancer vary markedly depending on their treatment strategy. Int J Cancer. 2016;139(4):749-53.

6. Koch-Gallenkamp L, Bertram H, Eberle A, Holleczek B, Schmid-Höpfner S, Waldmann A, et al. Fear of recurrence in long-term cancer survivors-Do cancer type, sex, time since diagnosis, and social support matter? Health Psychol. 2016;35(12):1329-33.

7. Lebel S, Tomei C, Feldstain A, Beattie S, McCallum M. Does fear of cancer recurrence predict cancer survivors' health care use? Supportive Care in Cancer. 2013;21(3):901-6.

8. Maguire R, Hanly P, Drummond FJ, Gavin A, Sharp L. Regret and fear in prostate cancer: The relationship between treatment appraisals and fear of recurrence in prostate cancer survivors. Psychooncology. 2017;26(11):1825-31.

9. Mahal BA, Chen MH, Bennett CL, Kattan MW, Sartor O, Stein K, et al. High PSA anxiety and low health literacy skills: drivers of early use of salvage ADT among men with biochemically recurrent prostate cancer after radiotherapy? Ann Oncol. 2015;26(7):1390-5.

10. Mehnert A, Lehmann C, Schulte T, Koch U. Presence of symptom distress and prostate cancer-related anxiety in patients at the beginning of cancer rehabilitation. Onkologie. 2007;30(11):551-6.

11. Mehta SS, Lubeck DP, Pasta DJ, Litwin MS. Fear of Cancer Recurrence in Patients Undergoing Definitive Treatment for Prostate Cancer: Results From CaPSURE. The Journal of Urology. 2003;170(5):1931-3.

12. Nilsson R, Næss-Andresen TF, Myklebust TÅ, Bernklev T, Kersten H, Haug ES. Fear of Recurrence in Prostate Cancer Patients: A Cross-sectional Study After Radical Prostatectomy or Active Surveillance. European Urology Open Science. 2021;25:44-51.

13. Roth AJ, Rosenfeld B, Kornblith AB, Gibson C, Scher HI, Curley-Smart T, et al. The Memorial Anxiety Scale for Prostate Cancer. Cancer. 2003;97(11):2910-8.

14. Sevier-Guy L-J, Ferreira N, Somerville C, Gillanders D. Psychological flexibility and fear of recurrence in prostate cancer. European Journal of Cancer Care.n/a(n/a):e13483.

15. Tavlarides AM, Ames SC, Diehl NN, Joseph RW, Castle EP, Thiel DD, et al. Evaluation of the association of prostate cancer-specific anxiety with sexual function, depression and cancer aggressiveness in men 1 year following surgical treatment for localized prostate cancer. Psychooncology. 2013;22(6):1328-35.

16. Touzani R, Mancini J, Troïan J, Bouhnik AD, Cussenot O, Gravis G, et al. Adaptation and validation of the memorial anxiety scale for prostate cancer (MAX-PC) in a sample of French men. J Patient Rep Outcomes. 2019;3(1):60.

17. Ussher JM, Perz J, Kellett A, Chambers S, Latini D, Davis ID, et al. Health-Related Quality of Life, Psychological Distress, and Sexual Changes Following Prostate Cancer: A Comparison of Gay and Bisexual Men with Heterosexual Men. J Sex Med. 2016;13(3):425-34.

18. van de Wal M, van Oort I, Schouten J, Thewes B, Gielissen M, Prins J. Fear of cancer recurrence in prostate cancer survivors. Acta Oncol. 2016;55(7):821-7.
