# Supplementary material for: Fear of cancer recurrence and PSA anxiety in patients with prostate cancer: a systematic review
Source: Support Care Cancer. 2022 Feb 1;30(7):5577–89. doi: 10.1007/s00520-022-06876-z (PMC9135793; doi:10.1007/s00520-022-06876-z)
Supplement: Supplementary file 6 — Supplementary file6 (DOCX 42 KB) [file 520_2022_6876_MOESM6_ESM.docx]

**Online resource 6: Risk of bias results for longitudinal studies**

| **Author** | **Group similarity** | **Exposure comparison** | **Exposure validity** | **Confounders identified** | **Strategies for confounders** | **Participants free of outcome** | **Outcome validity** | **Follow up time** | **Follow up complete** | **Strategies for follow up?** | **Statistical analysis** | **Score out of 9** | **Risk of bias** |
| --- | --- | --- | --- | --- | --- | --- | --- | --- | --- | --- | --- | --- | --- |
| Alvisi et al [1] | N/A | N/A | 1 | 0 | 0 | 1 | 1 | 1 | 1 | 0 | 1 | 6 | Moderate |
| Belizzi et al [2] | N/A | N/A | 1 | 1 | 1 | 1 | 1 | 1 | 0 | 0 | 1 | 7 | Moderate |
| Chambers et al [3] | N/A | N/A | 1 | 1 | 1 | 1 | 1 | 1 | 0 | 0 | 1 | 7 | Moderate |
| Chien et al [4] | 1 | N/A | 1 | 1 | 1 | 1 | 1 | 0 | 1 | N/A | 1 | 8 | Low |
| clark et al [5] | N/A | N/A | 1 | 1 | 1 | 1 | 1 | 1 | 1 | 0 | 1 | 8 | Low |
| Dowrick et al [6] | 1 | 1 | 1 | 0 | 0 | 1 | 1 | 1 | 1 | 0 | 1 | 8 | Moderate |
| Egger et al [7] | 1 | 0 | 1 | 0 | 0 | 1 | 1 | 1 | 1 | 0 | 1 | 7 | Moderate |
| Eisenberg et al [8] | N/A | N/A | 1 | 1 | 1 | 1 | 1 | 1 | 1 | 0 | 1 | 8 | Low |
| Ettridge et al [9] | N/A | N/A | 1 | 0 | 0 | 1 | 1 | 1 | 0 | 0 | 1 | 5 | Moderate |
| Götze et al [10] | N/A | N/A | 1 | 1 | 1 | 1 | 1 | 1 | 1 | 1 | 1 | 9 | Low |
| Naha et al [13] | N/A | N/A | 1 | 1 | 1 | 1 | 1 | 1 | 1 | 0 | 1 | 8 | Low |
| Meissner et al [12] | N/A | N/A | 1 | 1 | 1 | 1 | 1 | 1 | 1 | 0 | 1 | 8 | Low |
| Meissner et al [11] | N/A | N/A | 1 | 0 | 0 | 1 | 1 | 1 | 0 | 0 | 1 | 5 | Moderate |
| Parker et al [14] | N/A | N/A | 1 | 0 | 0 | 1 | 1 | 1 | 1 | 0 | 1 | 6 | Moderate |
| Savard et al [15] | N/A | N/A | 1 | 0 | 0 | 1 | 1 | 1 | 1 | 0 | 1 | 6 | Moderate |
| van den Bergh et al [16] | N/A | N/A | 1 | 0 | 0 | 1 | 1 | 0 | 1 | 0 | 1 | 5 | Moderate |
| Van stam et al [17] | N/A | N/A | 1 | 0 | 0 | 1 | 0 | 1 | 0 | 0 | 1 | 4 | High |
| Victorson et al [18] | N/A | N/A | 1 | 0 | 0 | 1 | 0 | 1 | 0 | 0 | 1 | 4 | High |
| Villa ET AL [19] | N/A | N/A | 1 | 0 | 0 | 1 | 1 | 1 | 0 | 0 | 1 | 5 | Moderate |
| Wilcox et al [20] | N/A | N/A | 1 | 0 | 0 | 1 | 1 | 0 | 0 | 0 | 1 | 4 | High |

References

1. Alvisi MF, Dordoni P, Rancati T, Avuzzi B, Nicolai N, Badenchini F, De Luca L, Magnani T, Marenghi C, Menichetti J, Silvia V, Fabiana Z, Roberto S, Riccardo V, Lara B, Prostate Cancer Multidisciplinary Clinic Working G (2020) Supporting Patients With Untreated Prostate Cancer on Active Surveillance: What Causes an Increase in Anxiety During the First 10 Months? Front Psychol 11: 576459

2. Bellizzi KM, Latini DM, Cowan JE, DuChane J, Carroll PR (2008) Fear of recurrence, symptom burden, and health-related quality of life in men with prostate cancer Urology 72: 1269-1273

3. Chambers SK, Ng SK, Baade P, Aitken JF, Hyde MK, Wittert G, Frydenberg M, Dunn J (2017) Trajectories of quality of life, life satisfaction, and psychological adjustment after prostate cancer Psychooncology 26: 1576-1585

4. Chien CH, Chuang CK, Liu KL, Wu CT, Pang ST, Chang YH (2018) Positive and negative affect and prostate cancer-specific anxiety in Taiwanese patients and their partners Eur J Oncol Nurs 37: 1-11

5. Clark JA, Talcott JA (2006) Confidence and uncertainty long after initial treatment for early prostate cancer: survivors' views of cancer control and the treatment decisions they made J Clin Oncol 24: 4457-4463

6. Dowrick AS, Wootten AC, Botti M (2018) Does partnership status affect the quality of life of men having robotic-assisted radical prostatectomy (RARP) for localised prostate cancer? Applied Nursing Research 42: 51-55

7. Egger SJ, Calopedos RJ, O'Connell DL, Chambers SK, Woo HH, Smith DP (2018) Long-term Psychological and Quality-of-life Effects of Active Surveillance and Watchful Waiting After Diagnosis of Low-risk Localised Prostate Cancer Eur Urol 73: 859-867

8. Eisenberg SA, Kurita K, Taylor-Ford M, Agus DB, Gross ME, Meyerowitz BE (2015) Intolerance of uncertainty, cognitive complaints, and cancer-related distress in prostate cancer survivors Psychooncology 24: 228-235

9. Ettridge K, Wright K, Smith D, Chambers S, Corsini N, Evans S, Moretti K, Roder D, Scuffham P, Miller C (2020) Measuring psychosocial outcomes of men living with prostate cancer: feasibility of regular assessment of patient-reported outcomes Eur J Cancer Care (Engl): e13393

10. Götze H, Taubenheim S, Dietz A, Lordick F, Mehnert-Theuerkauf A (2019) Fear of cancer recurrence across the survivorship trajectory: Results from a survey of adult long-term cancer survivors Psychooncology 28: 2033-2041

11. Meissner VH, Herkommer K, Marten-Mittag B, Gschwend JE, Dinkel A (2017) Prostate cancer-related anxiety in long-term survivors after radical prostatectomy J Cancer Surviv 11: 800-807

12. Meissner VH, Olze L, Schiele S, Ankerst DP, Jahnen M, Gschwend JE, Herkommer K, Dinkel A Fear of cancer recurrence and disease progression in long-term prostate cancer survivors after radical prostatectomy: A longitudinal study Cancer n/a

13. Naha U, Freedland SJ, Abern MR, Moreira DM (2020) The association of cancer-specific anxiety with disease aggressiveness in men on active surveillance of prostate cancer Prostate Cancer Prostatic Dis

14. Parker PA, Davis JW, Latini DM, Baum G, Wang X, Ward JF, Kuban D, Frank SJ, Lee AK, Logothetis CJ, Kim J (2016) Relationship between illness uncertainty, anxiety, fear of progression and quality of life in men with favourable-risk prostate cancer undergoing active surveillance BJU Int 117: 469-477

15. Savard J, Ivers H (2013) The evolution of fear of cancer recurrence during the cancer care trajectory and its relationship with cancer characteristics Journal of Psychosomatic Research 74: 354-360

16. van den Bergh RC, Essink-Bot ML, Roobol MJ, Schröder FH, Bangma CH, Steyerberg EW (2010) Do anxiety and distress increase during active surveillance for low risk prostate cancer? J Urol 183: 1786-1791

17. van Stam MA, Aaronson NK, Bosch J, Kieffer JM, van der Voort van Zyp JRN, Tillier CN, Horenblas S, van der Poel HG (2020) Patient-reported Outcomes Following Treatment of Localised Prostate Cancer and Their Association with Regret About Treatment Choices Eur Urol Oncol 3: 21-31

18. Victorson David E, Schuette S, Schalet Benjamin D, Kundu Shilajit D, Helfand Brian T, Novakovic K, Sufrin N, McGuire M, Brendler C (2016) Factors Affecting Quality of Life at Different Intervals After Treatment of Localized Prostate Cancer: Unique Influence of Treatment Decision Making Satisfaction, Personality and Sexual Functioning Journal of Urology 196: 1422-1428

19. Villa S, Repetto C, Rancati T, Avuzzi B, Catanzaro M, Marenghi C, Nicolai N, Stagni S, Maffezzini M, Salvioni R, Magnani T, Valdagni R, Bellardita L (2015) Prostate cancer-related anxiety: From enrolment to one year after the first re-biopsy Anticancer Research 35: 3671-3672

20. Wilcox CB, Gilbourd D, Louie-Johnsun M (2014) Anxiety and health-related quality of life (HRQL) in patients undergoing active surveillance of prostate cancer in an Australian centre BJU International 113: 64-68
